# Precise tuning of interlayer electronic coupling in layered conductive metal-organic frameworks

**DOI:** 10.1038/s41467-022-34820-6

**Published:** 2022-11-24

**Authors:** Yang Lu, Yingying Zhang, Chi-Yuan Yang, Sergio Revuelta, Haoyuan Qi, Chuanhui Huang, Wenlong Jin, Zichao Li, Victor Vega-Mayoral, Yannan Liu, Xing Huang, Darius Pohl, Miroslav Položij, Shengqiang Zhou, Enrique Cánovas, Thomas Heine, Simone Fabiano, Xinliang Feng, Renhao Dong

**Affiliations:** 1 Center for Advancing Electronics Dresden & Faculty of Chemistry and Food Chemistry, Technische Universität Dresden, Dresden, Germany; 2 Laboratory of Organic Electronics, Department of Science and Technology, Linköping University, Norrköping, Sweden; 3 Instituto Madrileño de Estudios Avanzados en Nanociencia (IMDEA Nanociencia), Madrid, Spain; 4 Central Facility for Electron Microscopy, Electron Microscopy of Materials Science, Universität Ulm, Ulm, Germany; 5 Institute of Ion Beam Physics and Materials Research, Helmholtz-Zentrum Dresden-Rossendorf, 01328 Dresden, Germany; 6 Dresden Center for Nanoanalysis (DCN), Center for Advancing Electronics Dresden (Cfaed), Technische Universität Dresden, Dresden, Germany; 7 Max Planck Institute of Microstructure Physics, 06120 Halle (Saale), Germany; 8 Key Laboratory of Colloid and Interface Chemistry of the Ministry of Education, School of Chemistry and Chemical Engineering, Shandong University, Jinan, 250100 China

**Keywords:** Electronic properties and materials, Two-dimensional materials, Metal-organic frameworks, Coordination polymers

## Abstract

Two-dimensional conjugated metal-organic frameworks (2D c-MOFs) have attracted increasing interests for (opto)-electronics and spintronics. They generally consist of van der Waals stacked layers and exhibit layer-depended electronic properties. While considerable efforts have been made to regulate the charge transport within a layer, precise control of electronic coupling between layers has not yet been achieved. Herein, we report a strategy to precisely tune interlayer charge transport in 2D c-MOFs via side-chain induced control of the layer spacing. We design hexaiminotriindole ligands allowing programmed functionalization with tailored alkyl chains (HATI_CX, X = 1,3,4; X refers to the carbon numbers of the alkyl chains) for the synthesis of semiconducting Ni_3_(HATI_CX)_2_. The layer spacing of these MOFs can be precisely varied from 3.40 to 3.70 Å, leading to widened band gap, suppressed carrier mobilities, and significant improvement of the Seebeck coefficient. With this demonstration, we further achieve a record-high thermoelectric power factor of 68 ± 3 nW m^−1^ K^−2^ in Ni_3_(HATI_C3)_2_, superior to the reported holes-dominated MOFs.

## Abstract (web summary)

Layered metal-organic frameworks attract interests for optoelectronics and spintronics. Here, the authors report a strategy to tune interlayer charge transport and thermoelectric properties via side-chain induced control of the layer spacing.

## Introduction

Electrically conductive metal-organic frameworks (MOFs), as an emerging class of electronic materials^1–3^, have recently drawn increasing attention and exhibited potential for broad applications, such as electrocatalysis^4–6^, energy storage^7–9^, optoelectronic devices^10–12^, chemiresistive sensors^13,14^, and thermoelectrics^15–17^. Linking *π*-conjugated ligands and metal ions through square-planar secondary building units (SBUs) can form a unique class of conductive MOFs, i.e., two-dimensional conjugated MOFs (2D c-MOFs), which show similar van der Waals structural features to graphite and other inorganic 2D crystals^3,18–22^. 2D c-MOFs comprise layer-stacked structures with in-plane *π*-extended conjugation and out-of-plane *π*-orbital overlap, thus allowing efficient transport of charge carriers within the 2D plane and along with the *π*-stacking direction^23^. These features enable 2D c-MOFs with enhanced electrical conductivities than conventional MOFs and charge mobility with band-like transport^24,25^. Besides the discovery of excellent charge transport in 2D c-MOFs, growing interest has been focused on the precise regulation of their (opto-)electronic properties (e.g., band gaps, carrier mobilities) through chemical design.

In the last decade, great efforts have been made to vary the charge transport properties via tuning the intralayer *π*-extended conjugation of 2D c-MOFs. This has been achieved by designing the topological networks and varying the chemical structures of the ligands (e.g., introducing heteroatoms or expanding the π-plane)^4,23,26,27^, as well as by tailoring the composition of the SBUs (e.g., metal ions and coordination functional groups)^28,29^. As a unique feature of layer-stacked van der Waals materials, the charge transport properties of 2D c-MOFs also strongly depend on the electronic coupling between layers^21,30,31^. However, little is known about the synthetic strategy to precisely control the interlayer electronic coupling in 2D c-MOFs.

**Fig. 1:**
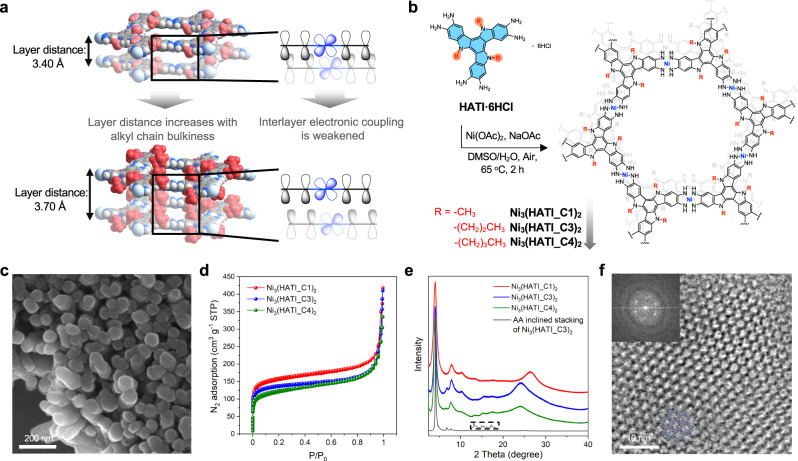
Design, synthesis, and structural elucidation of 2D c-MOFs. **a** Schematic diagram of the concept of the precise control of the interlayer electronic coupling. red balls: alkyl chains; gray balls: C; white balls: H; blue balls: N; cyan balls: Ni. **b** Synthesis of Ni_3_(HATI_CX)_2_ with different lengths of alkyl chains. **c** SEM images of Ni_3_(HATI_C3)_2_. **d** N_2_ adsorption isotherm of Ni_3_(HATI_CX)_2_. **e** XRD patterns for Ni_3_(HATI_C1)_2_, Ni_3_(HATI_C3)_2_, and Ni_3_(HATI_C4)_2_, and calculated PXRD patterns of Ni_3_(HATI_C3)_2_ with AA-inclined stacking modes. **f** HRTEM images of Ni_3_(HATI_C3)_2_, the inset is the related FFT analysis.

Herein, we report a strategy to precisely tune interlayer electronic coupling in 2D c-MOFs via side-chain-induced control of the layer spacing. We design a *π*-conjugated 2,3,7,8,12,13-hexaiminotriindole (HATI) ligand which enables the modification of alkyl chains with different steric bulkiness (methyl, *n*-propyl, and *n*-butyl groups, denoted as C1, C3, and C4, respectively) at nitrogen atoms of indole (Fig. 1a, b). Then, we synthesize a series of triindole-based 2D c-MOFs with the same SBUs but the tunable layer distance from 3.40 (C1) to 3.70 Å (C4). Theoretical calculation suggests that the insulated alkyl chains have no effect on the electronic structures within a 2D layer of MOFs; however, the electronic coupling between the layers and energy band dispersion are continuously weakened with increasing layer distance (Fig. 1a). The charge carrier dynamics studies demonstrate that the carrier mobility decreases gradually from 8.9 to 0.7 cm^2^ V^−1^ s^−1^ with the increase of the interlayer distance, which in turn leads to a decrease of the electrical conductivity from 1.1 to 0.09 S m^−1^ and an increase of the Seebeck coefficient from 76 to 424 μV K^−1^. As a result, a competitive balance between electrical conductivity and the Seebeck coefficient enables the Ni_3_(HATI_C3)_2_ to deliver a record *p*-type thermoelectric power factor of 68 ± 3 nW m^−1^ K^−2^ among the reported MOFs. Our work brings up a molecular design strategy for the synthesis of layer-stacked 2D c-MOFs toward tailored charge transport properties at the molecular level.

## Results

The methyl, *n*-propyl, and *n*-butyl groups are introduced into hexaiminotriindole ligands, named here as HATI-C1, HATI-C3, and HATI-C4, respectively (details shown in Supplementary Information). According to the calculation of surface charge distribution, the alkyl chains have negligible influence on the frontier orbital energy levels and the electrostatic potential distribution of hexaiminotriindole core (Supplementary Fig. S1). Reactions of HATI with Ni^2+^ ions in mixtures of dimethyl sulfoxide (DMSO) and water at 65 °C for 2 h led to the formation of polycrystalline 2D c-MOFs, Ni_3_(HATI_CX)_2_ (X = 1, 3, and 4, details seen in the Supplementary Information). The ratio of DMSO to water for MOF synthesis was decreased with the increase of the alkyl chain length due to the enhanced ligand solubility in DMSO (DMSO/H_2_O = 4.5/0.5 for Ni_3_(HATI_C1)_2_, DMSO/H_2_O = 3/2 for Ni_3_(HATI_C3)_2_ and Ni_3_(HATI_C4)_2_) (Supplementary Fig. S2).

Scanning electron microscopy (SEM) reveals that Ni_3_(HATI_C1)_2_, Ni_3_(HATI_C3)_2_, and Ni_3_(HATI_C4)_2_ powders consist of rod-like crystals (Fig. 1c and Supplementary Figs. S3, S4). The high-resolution analysis of the Ni(2*p*) of the X-ray photoelectron spectra (XPS) evidences the presence of Ni(II) in all 2D c-MOFs (Supplementary Fig. S5). In addition, Na^+^ peaks were not observed; the absence of extraneous cations suggested the neutral states of Ni_3_(HATI_CX)_2_. Fourier-transform infrared (FT-IR) spectroscopy of Ni_3_(HATI_CX)_2_ reveals the disappearance of the N-H stretching vibration band from the ligand HATI_CX (Supplementary Fig. S6), which further demonstrates the efficient coordination polymerization. Thermogravimetric analysis (TGA) indicates that all Ni_3_(HATI_CX)_2_ start desolvation over 100 °C and exhibit pronounced weight losses above 200 °C due to decomposition (Supplementary Fig. S7). N_2_ adsorption isotherms at 77 K presents the Brunauer−Emmett−Teller (BET) surface areas of 480, 413, and 385 m^2^ g^−1^ for Ni_3_(HATI_C1)_2_, Ni_3_(HATI_C3)_2_, and Ni_3_(HATI_C4)_2_, respectively (Fig. 1d and Supplementary Fig. S8). The surface areas of these 2D c-MOFs decrease with increasing the length of the alkyl chains grafted onto the pore walls.

Figure 1e exhibits the powder X-ray diffraction (PXRD) patterns of the Ni_3_(HATI_CX)_2_ under their optimal synthesis conditions. The major diffraction peaks resolved in the low-angle region (3.95°, 7.88°, 10.16°) are shared by the three resultant MOFs, which indicates that the in-plane crystalline ordering remains consistent with different alkyl chain lengths. The PXRD data manifest the interlayer spacing values of 3.40, 3.68, and 3.70 Å for the Ni_3_(HATI_C1)_2_, Ni_3_(HATI_C3)_2_, and Ni_3_(HATI_C4)_2_, respectively. The *π*-stacking distance gradually increases with the increase of the alkyl chain length from C1 to C4 in these 2D c-MOFs. Additionally, we performed statistical structural modeling with several possible interlayer arrangements using by density functional tight binding (DFTB) method (Fig. 1e)^32^. The AA-eclipsed stacking shows higher energy of 329 kJ mol^−1^ than AA-inclined stacking. And the experimental XRD pattern could be reproduced well with AA-inclined geometry, which is also energetically favorable (Fig. 1e, Supplementary Fig. S9, and Table S1). In combination with Pawley refinement results of Ni_3_(HATI_CX)_2_, all MOF samples fit for the triclinic unit cells (Supplementary Fig. S10). The experimental PXRD patterns exhibit the approximate width at half maximum, which suggests a similar crystallinity for these three samples. The local structures of three samples were further confirmed by high-resolution transmission electron microscopy (HRTEM). The fast Fourier-transform (FFT) analysis of HRTEM images of Ni_3_(HATI_CX)_2_ presents the honeycomb lattice with a lattice distance of 25.4 Å (Fig. 1f and Supplementary Fig. S11), which is consistent with the PXRD and calculated results.

**Fig. 2:**
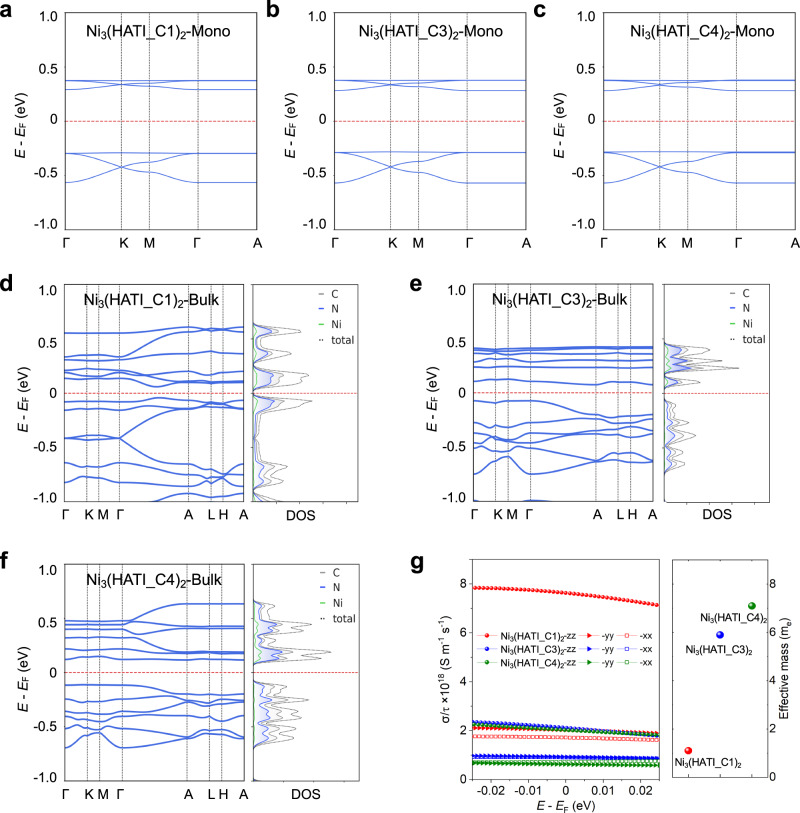
Theoretical calculation of electronic structures and conductivities for 2D c-MOFs. Modeling of the electronic structures of Ni_3_(HATI_CX)_2_. The calculated band structures of monolayer **a** Ni_3_(HATI_C1)_2_, **b** Ni_3_(HATI_C3)_2_, and **c** Ni_3_(HATI_C4)_2_. The calculated band structures and projected density of states (PDOS) of bulk **d** Ni_3_(HATI_C1)_2_, **e** Ni_3_(HATI_C3)_2_, and **f** Ni_3_(HATI_C4)_2_. **g** Calculated electrical conductivity (left) within the constant-relaxation-time approximation of the Boltzmann transport equation using the BoltzTraP2 code and average effective mass of 2D c-MOFs (right).

In order to gain an in-depth understanding of the effect of alkyl chain length on the electronic structures and charge transport properties of Ni_3_(HATI_CX)_2_, DFT calculation is performed to predict the energy band diagrams for single-layer and layer-stacked 2D c-MOFs. The surface charge distributions and electronic band structures for the Ni_3_(HATI_CX)_2_ monolayers are shown in Fig. 2a–c and Supplementary Fig. S15. In the conduction and valence bands of Ni_3_(HATI_CX)_2_, there is no charge distribution on the alkyl chains, which are “electrically insulating.” In Fig. 2a–c, the monolayers of Ni_3_(HATI_CX)_2_ with different length of alkyl chains display similar band structures with band gaps of 0.57 eV; therefore, the grafting of alkyl chains with varied lengths hardly influence the charge transport properties within the 2D plane in Ni_3_(HATI_CX)_2_. In band structures of layer-stacked Ni_3_(HATI_CX)_2_, we observe appreciable dispersion of the bands along the out-of-plane direction, which suggests that the interlayer coupling plays a critical role in the charge transport in bulk Ni_3_(HATI_CX)_2_ (Fig. 2d–f). The addition of various alkyl chains indirectly affects the out-of-plane electronic coupling (including HATI ligands and Ni[NH]_4_ nodes) by tuning the interlayer spacing (Fig. S13). Here, the interlayer energy bands near the Fermi level exhibit an average dispersion of 0.17, 0.12, and 0.09 eV for Ni_3_(HATI_C1)_2_, Ni_3_(HATI_C3)_2_, and Ni_3_(HATI_C4)_2_, respectively. According to the band structures, the bulk effective masses of Ni_3_(HATI_C1)_2_, Ni_3_(HATI_C3)_2_, and Ni_3_(HATI_C4)_2_ are calculated to be *m*_ave_* = 1.10*m*_0_, *m*_ave_* = 5.90*m*_0_, and *m*_ave_* = 7.10*m*_0_, respectively (Fig. 2g), indicating that the electronic coupling decreases in the order of alkyl chain length. The higher dispersion of the bands indicates lower effective mass and higher carrier mobility along the interlayer direction compared with the intralayer direction. In addition, the theoretical band gaps of Ni_3_(HATI_C1)_2_, Ni_3_(HATI_C3)_2_, and Ni_3_(HATI_C4)_2_ can be deduced as 0.09, 0.15, and 0.23 eV, respectively, which are enlarged with the increase of the interlayer spacing^33^. Figure 2g shows the plot of the conductivity at constant-relaxation time, which reveals that the difference between the interlayer conductivity and the intralayer one decreases gradually with the increase of the interlayer distance; the intralayer conductivity is lower than the interlayer one for all three cases. Ni_3_(HATI_C1)_2_ has significantly higher interlayer conductivity than the other two materials, further suggesting a greater contribution from the interlayer electronic coupling.

**Fig. 3:**
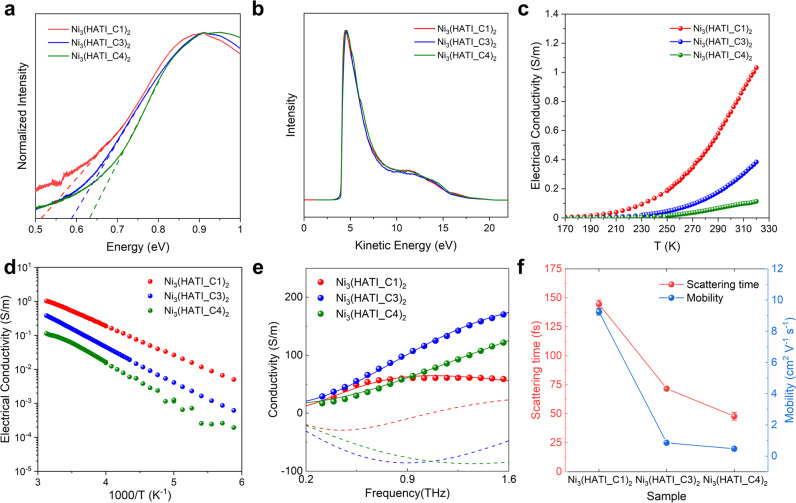
Optoelectronic and charge transport properties of 2D c-MOFs. **a** Vis−NIR absorption spectroscopy of Ni_3_(HATI_CX)_2_; **b** UPS results of Ni_3_(HATI_CX)_2_, the abscissa axis is kinetic energy; **c**, **d** variable-temperature electrical conductivities of Ni_3_(HATI_CX)_2_; **e** Frequency resolved conductivity for the three samples; **f** Scattering time and mobility of Ni_3_(HATI_CX)_2_.

We next measured the visible/near-infrared (vis−NIR) absorption spectra of these 2D c-MOFs to further understand the differences in their optoelectronic properties. The polaron (or radical) band of the Ni_3_(HATI_CX)_2_ powder sample emerges in the near-infrared region. The optical gaps estimated from the onset of the near-infrared region of Ni_3_(HATI_CX)_2_ reveal a continuous increase from 0.50 to 0.64 eV with a rise in interlayer spacing from 3.40 to 3.70 Å (Fig. 3a). This trend is consistent with the theoretical calculation results and can be attributed to the changes in the electronic coupling of the front molecular orbital^34–36^. The work functions of Ni_3_(HATI_CX)_2_ were further investigated by the ultraviolet photoelectron spectra (UPS, Fig. 3b). All Ni_3_(HATI_CX)_2_ exhibited the same work function of 4.0 eV, which indicates that the alkyl chain length hardly influences the carrier concentration (*n*) of these 2D c-MOFs due to the identical *π-d* conjugated structure.

The four-probe van der Pauw measurements under vacuum provide the room temperature electrical conductivities of the powder pellets as 1.1 ± 0.1 S m^−1^, 0.45 ± 0.07 S m^−1^, and 0.09 ± 0.02 S m^−1^ for Ni_3_(HATI_C1)_2_, Ni_3_(HATI_C3)_2_, and Ni_3_(HATI_C4)_2_, respectively (Fig. 3c). The electrical conductivities of Ni_3_(HATI_CX)_2_ decrease over one order of magnitude with the increase of the interlayer distance. As shown in Fig. 3c, all the samples present thermally-activated charge transport according to the variable-temperature (VT) conductivity measurements from 200 to 320 K. The hopping activation energies are 170, 209, and 220 meV for Ni_3_(HATI_C1)_2_, Ni_3_(HATI_C3)_2_, and Ni_3_(HATI_C4)_2_, respectively (Fig. 3d and Supplementary Fig. S14).

**Fig. 4:**
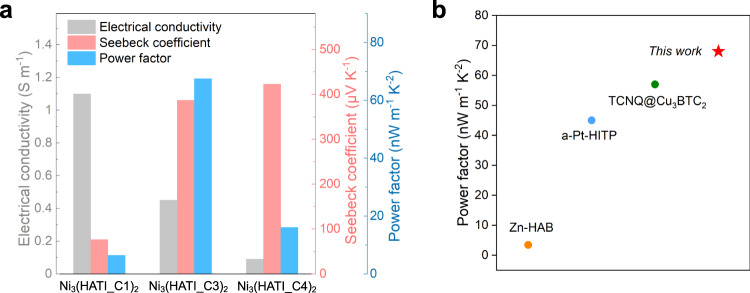
Seebeck coefficients and thermoelectric properties of 2D c-MOFs. **a** The thermoelectric properties (electrical conductivity, Seebeck coefficient, and power factor) of Ni_3_(HATI_CX)_2_ pellets measured under vacuum at 300 K; **b** Power factors of Ni_3_(HATI_C3)_2_ compared with other *p*-type thermoelectric MOFs.

We then performed terahertz time-domain spectroscopy (THz-TDS) measurements at room temperature under a dark environment to understand the charge transport properties of these 2D c-MOFs. Figure 3e reveals the real conductivity and imaginary components from the dark conductivity as a function of frequency for Ni_3_(HATI_C1)_2_, Ni_3_(HATI_C3)_2_, and Ni_3_(HATI_C4)_2_. As in other cases explored by THz spectroscopy^24^, the Drude−Smith model provides a good fit for the data (shown as solid lines in Fig. 3e, see methods in Supplementary Information). From the fit, we obtain the scattering rate (Fig. 3f) for the different samples; the free carrier scattering rate (*τ*) along with the average effective mass (m*, holes and electrons) provides an estimate for the carrier mobility as a function of alkyl length (i.e. interlayer distance). Thus, these 2D c-MOFs present carrier mobility of 9.2 ± 0.2, 0.9 ± 0.01, and 0.4 ± 0.03 cm^2^ V^−1^ s^−1^ for Ni_3_(HATI_C1)_2_, Ni_3_(HATI_C3)_2_, and Ni_3_(HATI_C4)_2_, respectively. Our results reveal that a shorter interlayer spacing between consecutive 2D c-MOF planes promotes charge carrier mobility and hence electrical conductivity. This conclusion agrees well with the trend observed in conductivities measured by electrical probes (Fig. 4a).

To further probe the influence of interlayer electronic coupling on charge transport in 2D c-MOFs, we investigated the Seebeck coefficient (*S*, thermal power) by a home-built steady-state measurement system at 300 K. In a typical procedure, the bulk sample Ni_3_(HATI_CX)_2_ was compressed into pellets with the size of 2 mm × 5 mm and the thickness of ~0.4 mm, and then was desolvated at 425 K for 2 h. Then the Seebeck coefficient was experimentally determined by measuring the electromotive force Δ*V* that develops across the material when temperature differences Δ*T* are present, as follows: *S* = Δ*V*/Δ*T*^37^. In theory, according to the Mott relation:

1 $$S=\frac{8{\pi }^{2}{k}_{B}^{2}}{3e{h}^{2}}{m}^{*}T{(\frac{\pi }{3n})}^{\frac{2}{3}}$$

the Seebeck coefficients are determined by the carrier concentration (*n*) and effective mass (m*) in periodic materials ($S\propto {m}^{*}/{n}^{\frac{2}{3}}$)^38^. As shown in Fig. 4a, all the MOF samples exhibit the positive Seebeck coefficients, suggesting that the electron-rich triindole units enable these 2D c-MOFs with holes-dominated transport. More importantly, the Seebeck coefficients are measured as 76.3 ± 2, 387.2 ± 2, 423.8 ± 1 μV K^−1^ for Ni_3_(HATI_C1)_2_, Ni_3_(HATI_C3)_2_, and Ni_3_(HATI_C4)_2_, respectively, which indicates a significant increase as the layer distance increases. The Seebeck coefficients of Ni_3_(HATI_C3)_2_ and Ni_3_(HATI_C4)_2_ are superior to those for the reported conductive MOFs (<200 μV K^−1^)^15–17^. The time-dependent evolution of Δ*V* at different ∆*T* suggests that ions do not contribute to the Seebeck coefficient values of Ni_3_(HATI_CX)_2_ (Supplementary Fig. S16). Furthermore, temperature-dependent Seebeck coefficient measurement shows a gradually decreasing trend with increasing temperature, supporting a thermally-activated charge transport in these 2D c-MOFs (Supplementary Fig. S17). Since the carrier concentration (*n*) in Ni_3_(HATI_CX)_2_ is independent of the alkyl chains length, the significant difference in Seebeck coefficients of Ni_3_(HATI_CX)_2_ samples should originate from the band structures and effective mass values, confirming the critical role of interlayer electronic coupling in charge transport in 2D c-MOFs. It is notable that a combination of the electrical conductivity and Seebeck coefficient yields a thermoelectric power factor (*PF*) as high as 68 ± 3 nW m^−1^ K^−2^ for Ni_3_(HATI_C3)_2_ at room temperature, which sets a record-high power factor value among the reported *p*-type MOFs (Fig. 5b and Supplementary Table S2)^15,17,39^.

In conclusion, we demonstrate the electronic coupling control in conductive 2D c-MOFs via alkyl chain substitution on *π*-conjugated ligands. The hexaiminotriindole ligands modified by alkyl chains with different lengths are synthesized and the layer spacing of the corresponding 2D c-MOFs can be precisely varied. As a result, the carrier mobility of these MOFs decreases with the increase of interlayer spacing, which further leads to the decrease of the conductivity and the increase of the Seebeck coefficients. This synthetic strategy of controlling the interlayer coupling of 2D c-MOFs will open the door to discover exotic physical properties of this class of van der Waals materials, like magic-angle graphene^40^.

## Methods

### Materials

The synthesis and characterization of 2,3,7,8,12,13-hexaiminotriindole (HATI) ligands with different steric bulkiness (methyl, *n*-propyl, and *n*-butyl groups, denoted as C1, C3, and C4, respectively) at nitrogen atoms of indole were shown in the Supplementary Information (Section S2 Synthetic procedures).

### Synthesis of Ni_3_(HATI-CX)_2_ 2D c-MOFs

Ni(OAc)_2_·4H_2_O (1.5 eq) and NaOAc (150 eq) in DMSO/H_2_O (3.5 mL, the ratio of DMSO/H_2_O was varied from 4.5:0.5 to 3:2) were preheated at 65 °C, to which was added a solution of 5 mg (1 eq.) of HATI-CX·6HCl in 1.5 mL of DMSO. This mixture was heated in a 10 mL open glass vial with stirring for 2 h at 65 °C. The resulting black powder was filtered, washed with a large amount of water and acetone, and dried under vacuum at room temperature.

### AC conductivity measurements by THz

For the THz-TDS measurements of the MOFs, we have measured the THz signals in a box purged with N_2_ to avoid water absorption of the THz light. We have measured the reference (THz pulse as it is passing through a mask where we placed the MOF samples). Secondly, we measured the samples deposited on the mask (Supplementary Fig. S18). We have tried to resolve the photoconductivity under 400 nm of optical excitation without acquiring any signal from OPTP (Optical pump Terahertz probe).

An optical rectification method was used to generate THz light on a ZnTe crystal slab with a Ti: Sapphire laser amplifier, which provided 150 fs laser pulses at 775 nm, with a repetition rate of 1 kHz. The measurement was carried out via electro-optical sampling on an identical ZnTe crystal.

The experimental results have been fitted with the Drude-Smith model (2)

2 $${\sigma }_{{DS}}=\frac{{\omega }_{p}^{2}{\varepsilon }_{0}\tau }{1-i\omega \tau }\left(1+\frac{c}{1-i\omega \tau }\right)$$

Localization effects are described by this model, and it helps to understand how long-range carrier transport is suppressed. There are three parameters, ${\omega }_{p}$, $\tau$, and $c$, which correspond to the plasma frequency (${\omega }_{p}^{2}=N{e}^{2}/{\varepsilon }_{0}{m}^{*}$, with *N* the charge carrier density, $e$ the electron charge and ${m}^{*}$ the effective mass), scattering time, and confinement parameters, respectively. The confinement parameter describes the backscattering coefficient, ranging from 0 (pure Drude behavior) to −1 (total charge carrier localization and null conductivity in the DC limit). The Drude-Smith model is able to offer carrier mobility value as $\mu=(e{\tau }_{s}/{m}^{*})(1+c)$. A Drude-Smith response indicates that charge carriers are limited in their long-range drift by the restoring force.

### Seebeck coefficient measurements

Seebeck coefficient measurements were performed inside a nitrogen-filled glovebox using a Keithley 4200 SCS semiconductor characterization system. The sample preparation was the same as in the case of the electrical conductivity measurements. Before measurement, the pellets were heated at 150 °C in a vacuum for 2 h for complete desolvation and excluding any ionic contribution to Seebeck coefficient values. The channel length/width is 0.5 mm/2 mm for Seebeck coefficient characterizations.

### Modeling and first-principles calculations

The geometry optimizations were calculated using the density functional tight binding (DFTB) by the ADF-DFTB code with the trans3d-0-1 parameter set and UFF dispersion correction. Band structures and density of states (DOS) calculations were performed with an implement of FIH-aims with a TIER1 basis set under 3 × 3 × 6. Herd−Scuseria−Ernzerhof hybrid functional (HSE06) was used, which can provide a more precise band gap than GGA and LDA. Many-body method was used to correct the long-range van der Waals dispersion in the band structure calculation.

### Reporting Summary

Further information on research design is available in the Nature Portfolio Reporting Summary linked to this article.

## Supplementary information

Supplementary Information

Lasing Reporting Summary

## Data Availability

The authors declare that all relevant data were available in this paper and its Supplementary Information and that data supporting the results of this study are available from corresponding authors upon reasonable request.
