# Supplementary material for: Precise tuning of interlayer electronic coupling in layered conductive metal-organic frameworks
Source: Nat Commun. 2022 Nov 24;13:7240. doi: 10.1038/s41467-022-34820-6 (PMC9700716; doi:10.1038/s41467-022-34820-6)
Supplement: Supplementary file 1 — Supplementary Information [file 41467_2022_34820_MOESM1_ESM.pdf]

Supplementary Information

Precise tuning of interlayer electronic coupling in layered conductive metal-organic frameworks

Y. Lu et al.

Table of Contents:

Supplementary Materials
Supplementary Methods
Supplementary Figures
Supplementary Tables

Supplementary Materials

All solvents, reagents and chemicals were purchased from commercial suppliers, such as Sigma-Aldrich, TCI and ABCR GmbH. and used without further purification unless specially addressed.

Supplementary Methods

Nuclear magnetic resonance (NMR) spectroscopy was recorded on Bruker AV-II 300 spectrometer operating at 300.1 MHz and Bruker AV-III 600 spectrometer operating at 600 MHz for ^{1}H NMR and 151 MHz for ^{13}C NMR. Chemical shifts are given in ppm relative to TMS.

Fourier transform infrared (FT-IR) spectroscopy was performed on a Bruker Optics ALPHA-E spectrometer with a universal Zn-Se ATR (attenuated total reflection) accessory in the 400−4000 cm^{-1}.

High-resolution MALDI-TOF mass spectrometry was recorded on a Bruker Autoflex Speed MALDI TOF MS (Bruker Daltonics, Bremen, Germany) with trans-2-[3-(4-tert-Butylphenyl)-2-methyl-2-propenylidene] malononitrile (DCTB) as the matrix. The instrument is equipped with an Agilent Series 1200 HPLC binary pump, and Autosampler, using Mass Hunter software.

Ultraviolet-Visible (UV/vis) absorption was measured on an Agilent Cary 5000 UV-VIS-NIR spectrophotometer at room temperature.

Powder X-ray diffraction (PXRD) patterns were obtained on an X-ray diffractometer (Aeris Research Edition, Malvern Panalytical Company) using Cu-Kα radiation (λ = 0.15418 nm) at 40 kV and 15 mA at room temperature. The as-obtained powder samples were measured in reflection geometry.

Scanning electron microscopy (SEM) were recorded on Zeiss Gemini S4500; **Transmission electron microscope (TEM)** were recorded on JEOL JEM F200.

Thermal gravimetric analysis (TGA) was characterized using a Thermal gravity analyses (TGA) were carried out on a TA Instrument Q600 analyzer under N_2 atmosphere with a heating rate of 5 ^{o}C/min in ceramic crucible. Before the measurement, the powder sample was treated in a supercritical CO_2 dryer for 4 hours, and then activated at 90 °C overnight. And the *in-situ* activation (100 °C for 1 h) was conducted during the TGA measurement.

Gas adsorption

Nitrogen sorption isotherms were measured on a BELSORP adsorption analyzer at liquid nitrogen temperature, and the surface area was calculated based on the adsorption curve according to the Brunauer-Emmett-Teller (BET) theory. Nitrogen sorption measurements were conducted at 77 K on a Quantachrome volumetric analyser. Before

the measurement, the powder sample was treated in a supercritical CO_2 dryer for 4 hours, and then activated at 90 °C overnight.

X-ray photoelectron spectroscopy (XPS) and ultraviolet photoelectron spectroscopy (UPS) were conducted on a Kratos AXIS Supra/Ultra Photoelectron Spectrometer under an ultrahigh vacuum of about 3×10^{-9} Torr with an unfiltered He I gas discharge lamp source (21.22 eV) and a monochromatic Al Kα source (1486.7 eV, θ = 90°, operated at 14 kV and 15 mA), respectively. The instrumental energy resolution for UPS and XPS were 0.1 eV and 0.5 eV, respectively. For sample preparation, all films were deposited on heavily doped n-type Si wafers in a N_2 glove box and transferred through a transport system without air exposure into the spectrometer analysis chamber. The Fermi level could be calculated from the secondary electron cutoff region (SECO); and the energy difference between the Fermi level and the HOMO level could be obtained from the onset of the low binding energy region of the highest occupied molecular orbital (HOMO).

Electrical conductivity

The pressed pellets were prepared by adding 8 mg samples onto a polymer film in a split sleeve pressing under the pressure at room temperature. The pellets were heated at 150 ^{o}C in vacuum for 2 hours for complete desolvation, which was consistent with the Seebeck coefficients measuremnts. Then the pressed pellets were taken out and the thickness was measured. Then, four probes of silver wires were placed onto the top of the pressed pellets using conductive silver plastic. The probe was transferred onto the probe station. Then, the device was kept in air for 1 h to keep the complete drying of the paste. The electrical conductivities of the samples in this work were determined in the van der Pauw geometries using a commercial Lakeshore Hall System (9700A). Activation energy was obtained through Arrhenius equation: σ (T) = $\sigma_0 \exp(-E_a/k_BT)$, σ_0 is the pre-factor and k_B is the Boltzmann's constant. And the influences on electrical conductivities of the activation treatment in these samples were summarized in Table S3.

THz measurements

For the THz-TDS measurements of the MOF we have measured the THz signals in a box purged with N_2 to avoid water absorption of the THz light. We have measured the reference (THz pulse as it is passing through a mask where we placed the MOF samples). Secondly, we have measured the samples deposited on the mask (Fig. S17). We have tried to resolve the photoconductivity under 400 nm of optical excitation without acquiring any signal from OPTP (Optical pump Terahertz probe).

An optical rectification method was used to generate THz light on a ZnTe crystal slab with a Ti: Sapphire laser amplifier, which provided 150 fs laser pulses at 775 nm, with a repetition rate of 1KHz. The measurement was carried out via electro-optical sampling on an identical ZnTe crystal.

The experimental results have been fitted with the Drude-Smith model (eq. 1)[1-2]

$$\sigma_{DS} = \frac{\omega_p^2 \varepsilon_0 \tau}{1 - i\omega\tau}\left(1 + \frac{c}{1 - i\omega\tau}\right) \text{ (eq. 1)}$$

Localization effects are described by this model, and it helps to understand how long-range carrier transport is suppressed. There are three parameters, ω_p, τ and c, which correspond to the plasma frequency ($\omega_p^2 = N e^2/\varepsilon_0 m^*$, with N the charge carrier density, e the electron charge and m^* the effective mass), scattering time, and confinement parameters, respectively. The confinement parameter describes the backscattering coefficient, ranging from 0 (pure Drude behavior) to -1 (total charge carrier localization and null conductivity in the DC limit). The Drude-Smith model is able to offer carrier mobility value as $\mu = (e\,\tau_s/m^*)(1 + c)$. A Drude-Smith response indicates that charge carriers are limited in their long-range drift by the restoring force.

Seebeck coefficients

Seebeck coefficient measurements were performed inside a nitrogen-filled glovebox using a Keithley 4200 SCS semiconductor characterization system.[3] The sample preparation was same with electrical conductivity measurements. Before measurement, the pellets were heated at 150 ^{o}C in vacuum for 2 hours for complete desolvation and excluding the ion contribution to Seebeck coefficients.[4] The channel length/width is 0.5 mm/2 mm for Seebeck coefficient characterizations.

Pawley refinement

The unit cells of the models were refined in the 2θ range 2.5-40° with the experimentally obtained PXRD pattern of **Ni_3(HANT-C1)$_2$**, **Ni_3(HANT-C3)$_2$,** and **Ni_3(HANT-C4)$_2$** MOFs in the Reflex module of the BIOVA Materials Studio 2017 (17.1.0.48. Copyright © 2016 Dassault Systèmes), with fixed atom coordinates. The obtained structural models were checked for bond length and bond angle consistency in the structure. The Pseudo-Voigt profile function was used for whole profile fitting during the refinement processes. The final Rwp and Rp values 3.83 % and 2.76 % for **Ni_3(HANT-C1)$_2$**, 5.43 % and 3.86 % for **Ni_3(HANT-C3)$_2$**, and 2.61 % and 2.95 % for **Ni_3(HANT-C4)$_2$**, respectively was obtained after the Pawley refinement.

Modeling and First-principles calculations

The geometry optimizations were calculated using the density functional tight binding (DFTB)[5] by the ADF-DFTB code[6] with the trans3d-0-1 parameter set[7] and UFF dispersion correction[8]. Band structures and density of states (DOS) calculations were performed with implement of FIH-aims[9] with TIER1 basis set under 3×3×6. Herd−Scuseria−Ernzerhof hybrid functional (HSE06)[10] was used, which can provide more precise band gap than GGA and LDA. Many-body method was used to correct the long-range van der Waals dispersion in the band structure calculation.[11]

The electrical conductivity calculations were performed in the Vienna Ab initio Simulation Package (VASP) with the optimized structure.[12,13] The electronic exchange-correlation functional was treated by the Perdew, Burke and Ernzerhof (PBE) with D3BJ dispersion correction.[14,15] The energy cutoff of the plane waves was set to 350 eV. The energy convergence criterion in the self-consistent calculations was set to 10^{-6}

eV. A Gamma-centered k-point mesh with a grid of 3×3×6 for geometry optimization.

Synthetic procedures

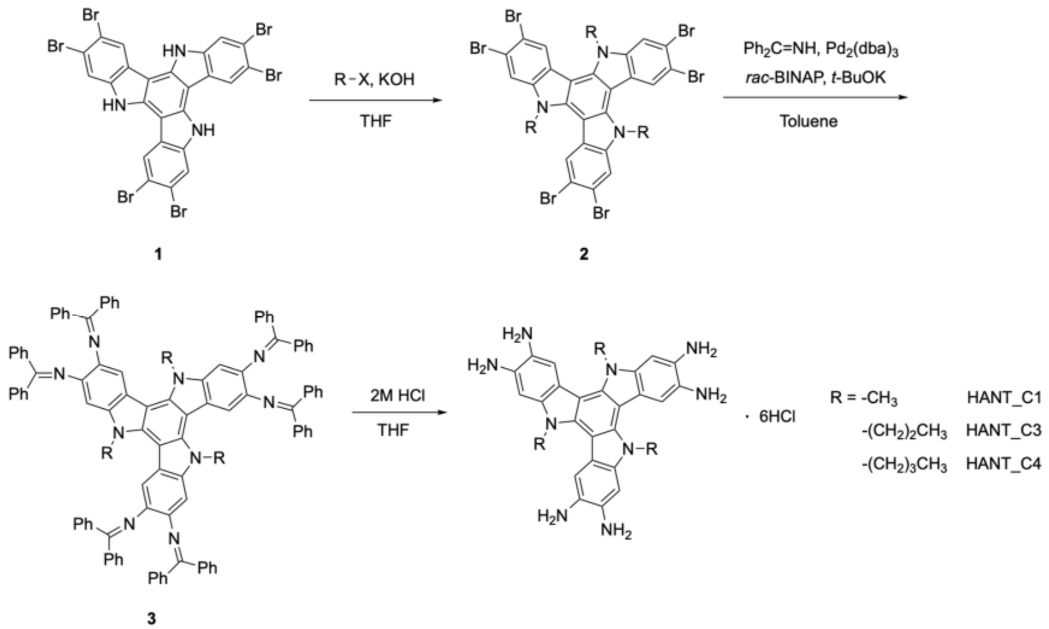

General Procedure:

Compound 2: A mixture of **1** (712 mg, 0.87 mmol) and KOH (487 mg, 8.7 mmol) in THF (20 mL) was heated under reflux for 30 min. 1-Halogenated alkanes (CH_3I, C_2H_5Br, C_3H_7Br, C_4H_9Br) (4.5 eq) was then added and the mixture was heated under reflux for 12 h. Thereafter, the solvent was evaporated under vacuum. The crude products were washed with water and acetone, dried under vacuum. The compounds **2-C1** and **2-C3** are difficult to be further purified due to their low solubility. The **2-C4** was purified by column chromatography on silica gel, eluting with CH_2Cl_2/EtOAc (5:1, v/v), to give the product as a light yellow solid in 70% yield. ^{1}H NMR (300 MHz, Chloroform-*d*) δ 8.09 (s, 1H), 7.62 (s, 1H), 4.32 – 4.06 (m, 2H), 1.76 (tt, *J* = 11.7, 7.0 Hz, 2H), 1.28 (h, *J* = 7.3 Hz, 2H), 0.91 (t, *J* = 7.3 Hz, 3H). ^{13}C NMR (76 MHz, $CDCl_3$) δ 140.16, 138.78, 125.08, 122.99, 118.50, 115.23, 114.67, 101.39, 46.82, 32.14, 19.81, 13.80. $C_{27}H_{15}Br_6N_3$ (**2-C1**)[M+] Exact Mass = 860.63, MS (MALDI-TOF) = 860.355; $C_{33}H_{27}Br_6N_3$ (**2-C3**)[M+] Exact Mass = 944.72, MS (MALDI-TOF) = 944.630; $C_{36}H_{33}Br_6N_3$ (**2-C4**)[M+] Exact Mass = 986.77, MS (MALDI-TOF) = 986.744.

Compound 3** and **HATI-CX: To a dry round bottom flask was added $Pd_2(dba)_3$ (0.24 eq), *rac*-BINAP (0.36 eq) and dry toluene (20 mL). The mixture was degassed by three freeze–pump–thaw cycles and heated at 110 °C for 30 min. The solution was then cooled to room temperature and compound **2** (1 eq), sodium tert-butoxide (7.8 eq), and benzophenone imine (7.8 eq) were added. The mixture was degassed again with three freeze–pump–thaw cycles and heated to 110 °C overnight. After cooling to room temperature, the solution was directly subject to a short silica gel column and flushed with eluent (Hexane (Hex):ethyl acetate (EA) = 5:1). The collected orange part was

evaporated to dryness in a single neck round bottom flask and directly used without further purification. $C_{105}H_{75}N_9$ (**2-C1**)[M+] Exact Mass = 1461.61, MS (MALDI-TOF) = 1461.57; $C_{111}H_{87}N_9$ (**2-C3**)[M+] Exact Mass = 1545.71, MS (MALDI-TOF) = 1545.68; $C_{114}H_{93}N_9$ (**2-C4**)[M+] Exact Mass = 1587.76, MS (MALDI-TOF) = 1578.72. Into the round bottom flask with the crude product **3** (200 mg) was added THF (10 mL) and the mixture was purged with nitrogen. 2.4 M hydrochloric acid (2 mL) was added dropwise and the mixture was stirred for another 30 min. The muddy product was then filtered off by suction filtration and washed thoroughly with THF. The collected HATI-CX was dried under vacuum and directly used for the next step without any further purification (75-85% yield based on compound 2). **HATI-C1**: ^{1}H NMR (300 MHz, DMSO-d_6) δ 8.50 (s, 1H), 7.15 (s, 1H), 4.04 (s, 3H). **HATI-C3**: ^{1}H NMR (300 MHz, DMSO-d_6) δ 8.24 (s, 1H), 7.40 (s, 1H), 4.72 (t, *J* = 7.0 Hz, 2H), 1.73 (q, *J* = 7.3 Hz, 2H), 0.53 (t, *J* = 7.4 Hz, 3H). **HATI-C4**: ^{1}H NMR (300 MHz, DMSO-d_6) δ 8.30 (s, 1H), 7.39 (s, 1H), 4.75 (s, 2H), 1.59 (s, 2H), 0.77 (s, 2H), 0.51 (d, *J* = 7.9 Hz, 3H). $C_{27}H_{27}N_9$ (**HATI-C1**)[M+] Exact Mass = 477.24, MS (MALDI-TOF) = 477.46; $C_{33}H_{39}N_9$ (**HATI-C3**)[M+] Exact Mass = 561.33, MS (MALDI-TOF) = 561.40; $C_{36}H_{45}N_9$ (**HATI-C4**)[M+] Exact Mass = 603.38, MS (MALDI-TOF) = 603.90.

Ni_3(HATI-CX)$_2$ synthesis:

$Ni(OAc)_2 \cdot 4H_2O$ (1.5 eq) and NaOAc (150 eq) in DMSO/H_2O (total 3.5 mL, the detailed ratio was according to the optimal PXRD patterns) (Fig. S2 and S19) were preheated at 65 °C, to which was added a solution of 5 mg (1 eq.) of HATI·6HCl in 1.5 mL of DMSO. Typically the total ratio of DMSO:H_2O is 4.5:0.5 for Ni_3(HATI-C1)$_2$, 3.5:1.5 for Ni_3(HATI-C2)$_2$, 3:2 for Ni_3(HATI-C3)$_2$, Ni_3(HATI-C4)$_2$, and Ni_3(HATI-C8)$_2$. This mixture was heated in a 10 mL open glass vial with stirring for 2 hours at 65 °C. The resulting black powder was filtered, and washed with large amount of water and acetone, and dried under vacuum at room temperature. Elemental analysis for Ni_3(HATI-C1)$_2$ ($Ni_3(C_{27}H_{21}N_9)_2$): Calculated: C: 57.95%; H: 3.78%; N: 22.53%. Found: C: 57.12%; H: 3.97%; N: 21.87%; Elemental analysis for Ni_3(HATI-C3)$_2$ ($Ni_3(C_{33}H_{33}N_9)_2$): Calculated: C: 61.57%; H: 5.17%; N: 19.58%. Found: C: 60.93%; H: 5.32%; N: 19.01%; Elemental analysis for Ni_3(HATI-C4)$_2$ ($Ni_3(C_{36}H_{39}N_9)_2$): Calculated: C: 63.05%; H: 5.73%; N: 18.38%. Found: C: 62.77%; H: 6.01%; N: 18.02%.

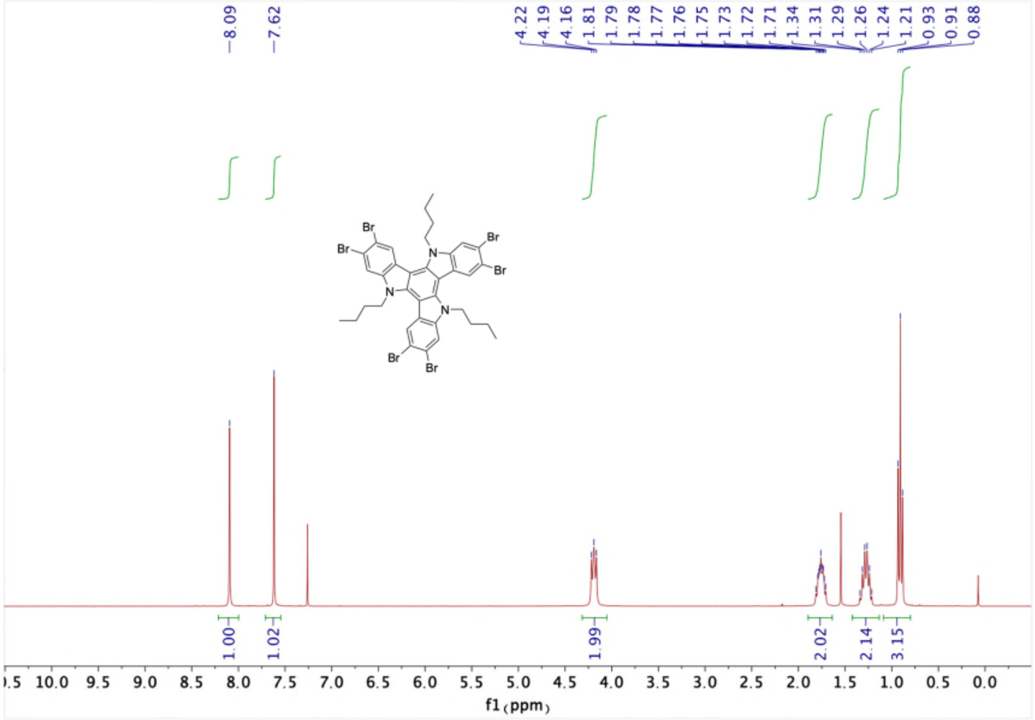
8.09
7.62
4.22
4.19
4.16
1.81
1.79
1.78
1.77
1.76
1.75
1.73
1.72
1.71
1.34
1.31
1.29
1.26
1.24
1.21
0.93
0.91
0.88
Br
Br
Br
Br
Br
Br
N
N
N
1.00
1.02
1.99
2.02
2.14
3.15
10.0 9.5 9.0 8.5 8.0 7.5 7.0 6.5 6.0 5.5 5.0 4.5 4.0 3.5 3.0 2.5 2.0 1.5 1.0 0.5 0.0
f1 (ppm)

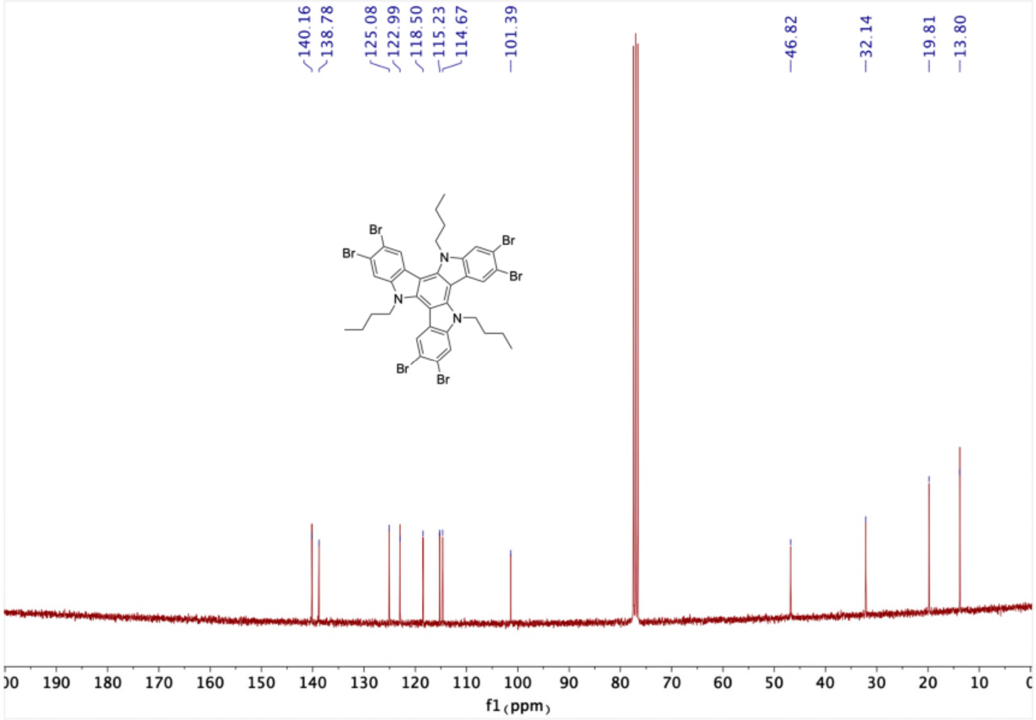
140.16
138.78
125.08
122.99
118.50
115.23
114.67
101.39
46.82
32.14
19.81
13.80
Br
Br
Br
Br
Br
Br
N
N
N
190 180 170 160 150 140 130 120 110 100 90 80 70 60 50 40 30 20 10
f1 (ppm)

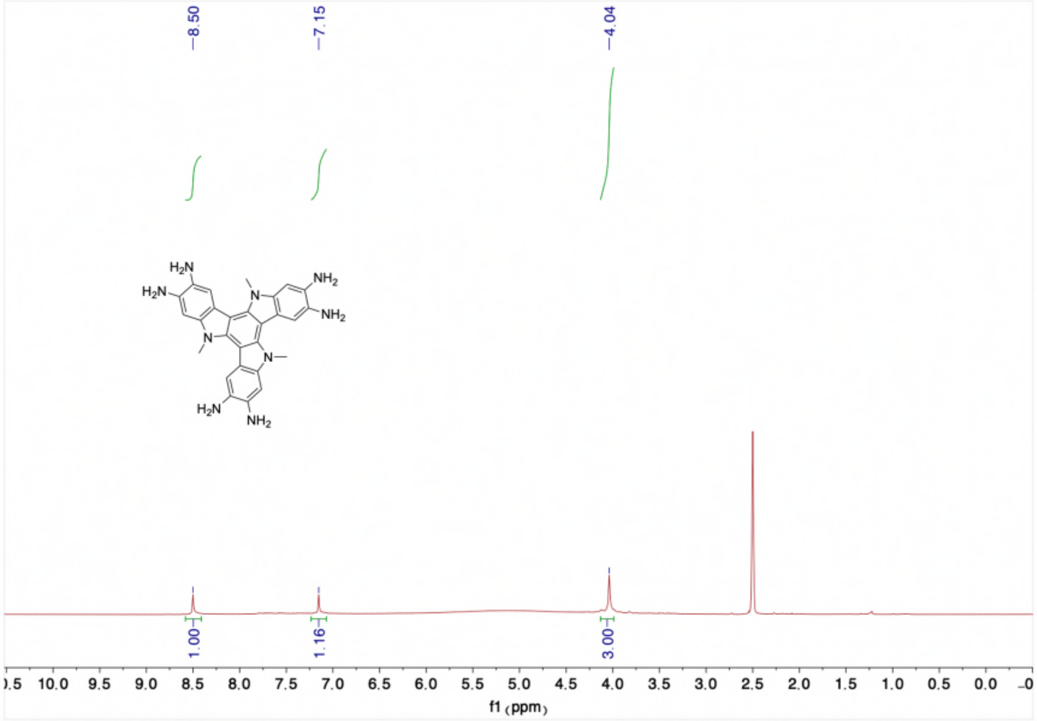
8.50
7.15
4.04
H2N
H2N
NH2
NH2
N
N
N
H2N
NH2
1.00
1.16
3.00
10.0 9.5 9.0 8.5 8.0 7.5 7.0 6.5 6.0 5.5 5.0 4.5 4.0 3.5 3.0 2.5 2.0 1.5 1.0 0.5 0.0
f1 (ppm)

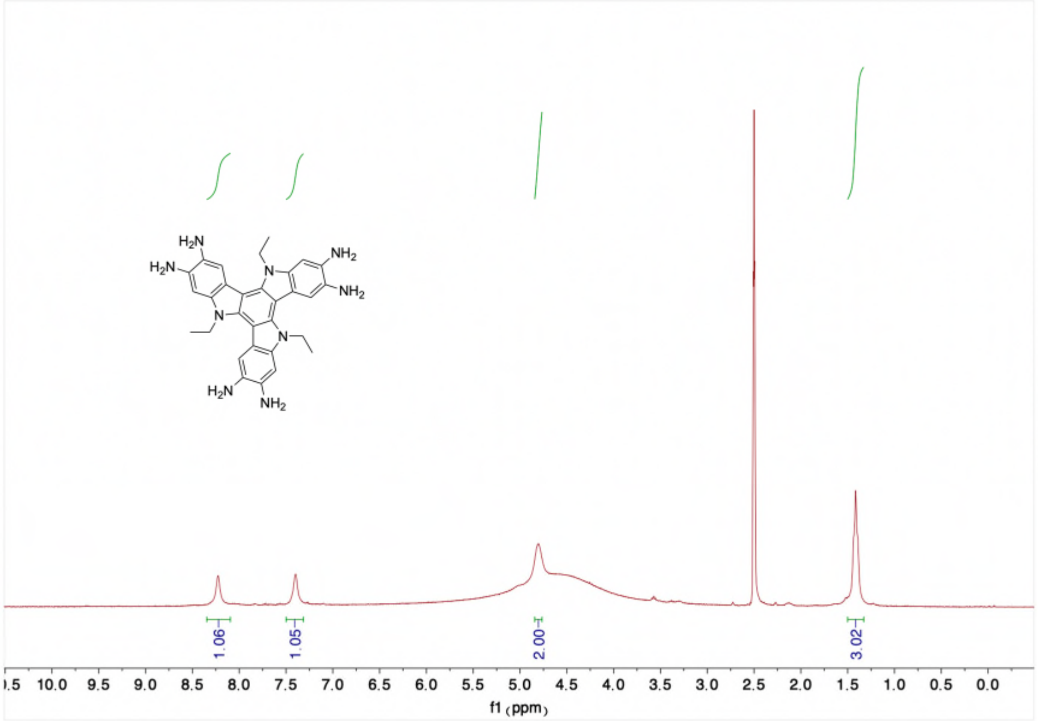
H2N
H2N
NH2
NH2
N
N
N
H2N
NH2
1.06
1.05
2.00
3.02
10.0 9.5 9.0 8.5 8.0 7.5 7.0 6.5 6.0 5.5 5.0 4.5 4.0 3.5 3.0 2.5 2.0 1.5 1.0 0.5 0.0
f1 (ppm)

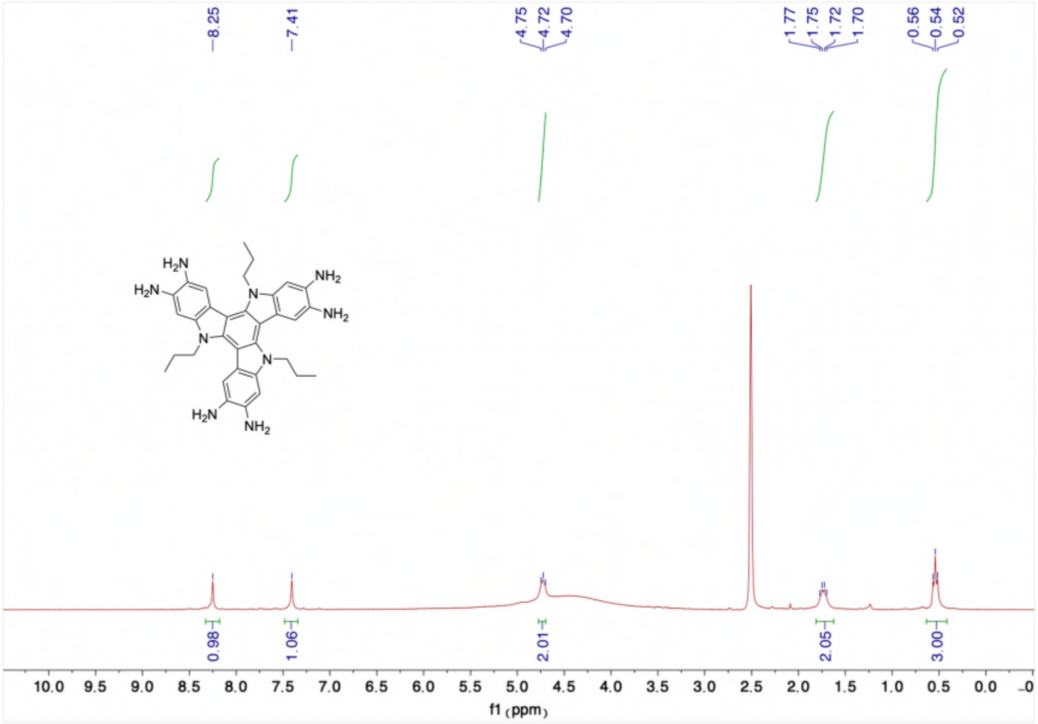
8.25
7.41
4.75
4.72
4.70
1.77
1.75
1.72
1.70
0.56
0.54
0.52
H2N
H2N
N
NH2
NH2
N
N
H2N
NH2
0.98
1.06
2.01
2.05
3.00
10.0 9.5 9.0 8.5 8.0 7.5 7.0 6.5 6.0 5.5 5.0 4.5 4.0 3.5 3.0 2.5 2.0 1.5 1.0 0.5 0.0 -0
f1 (ppm)

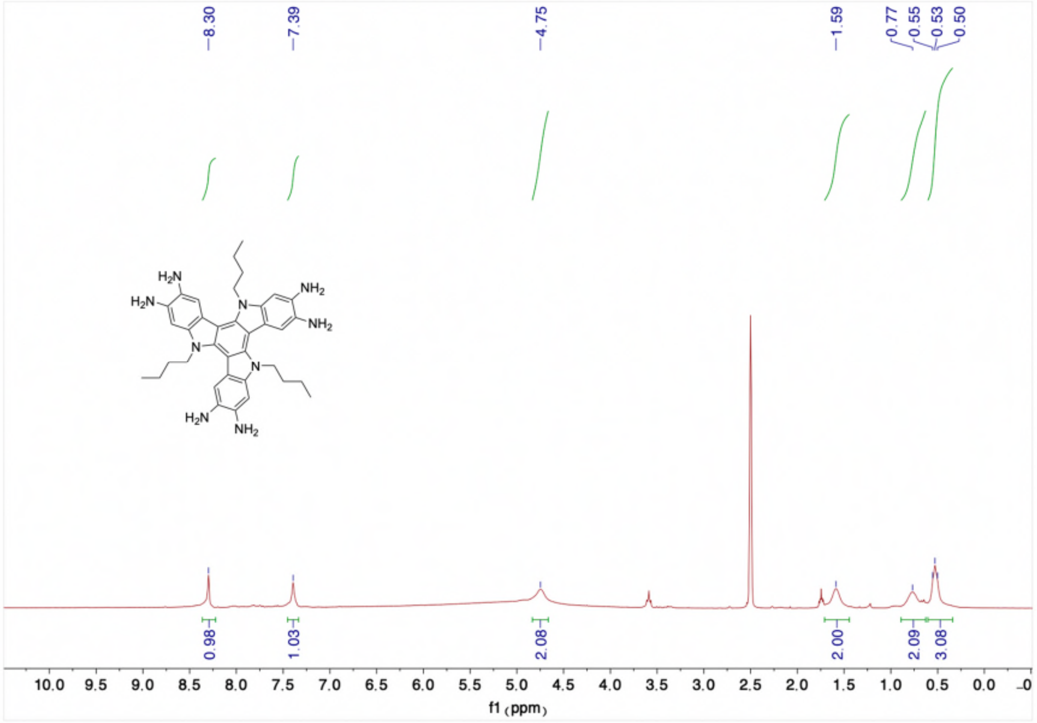
8.30
7.39
4.75
1.59
0.77
0.55
0.53
0.50
H2N
H2N
N
NH2
NH2
N
N
H2N
NH2
0.98
1.03
2.08
2.00
2.09
3.08
10.0 9.5 9.0 8.5 8.0 7.5 7.0 6.5 6.0 5.5 5.0 4.5 4.0 3.5 3.0 2.5 2.0 1.5 1.0 0.5 0.0 -0
f1 (ppm)

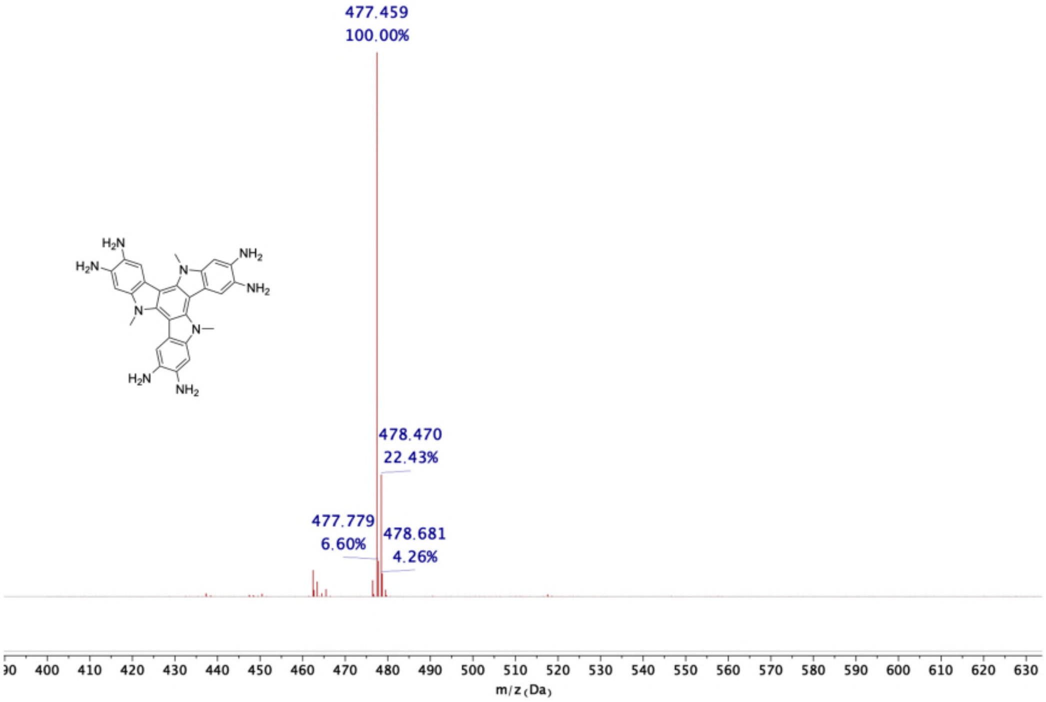
477.459
100.00%
478.470
22.43%
477.779
6.60%
478.681
4.26%
H2N
H2N
NH2
NH2
N
N
N
H2N
NH2
m/z (Da)

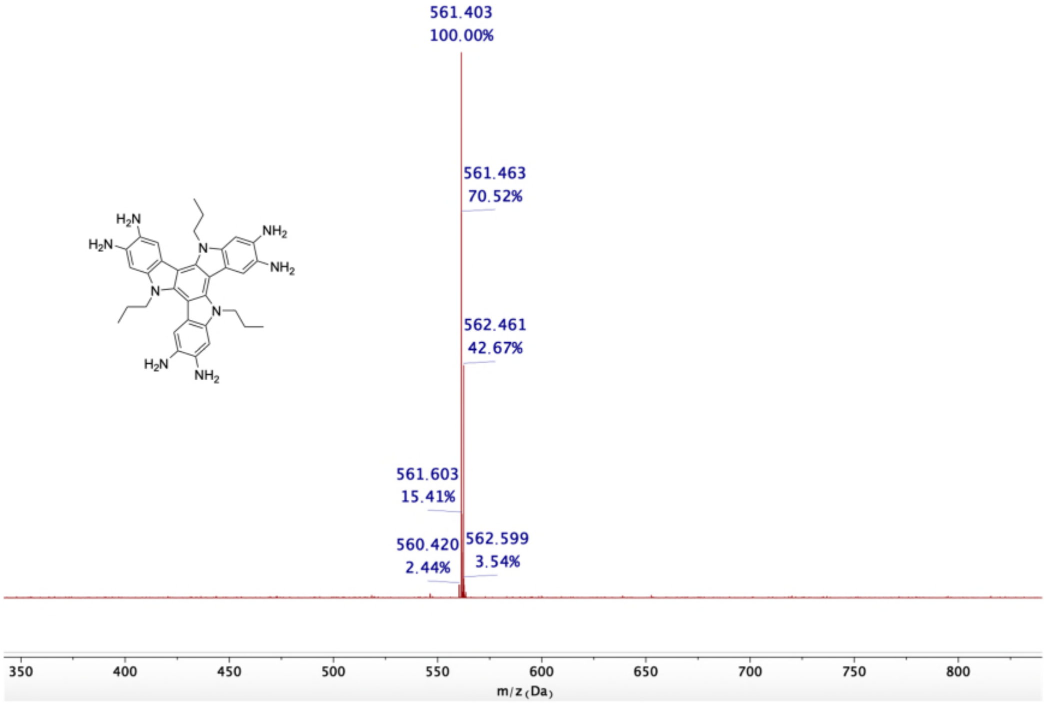
561.403
100.00%
561.463
70.52%
562.461
42.67%
561.603
15.41%
560.420
2.44%
562.599
3.54%
H2N
H2N
NH2
NH2
N
N
N
H2N
NH2
350
400
450
500
550
600
650
700
750
800
m/z (Da)

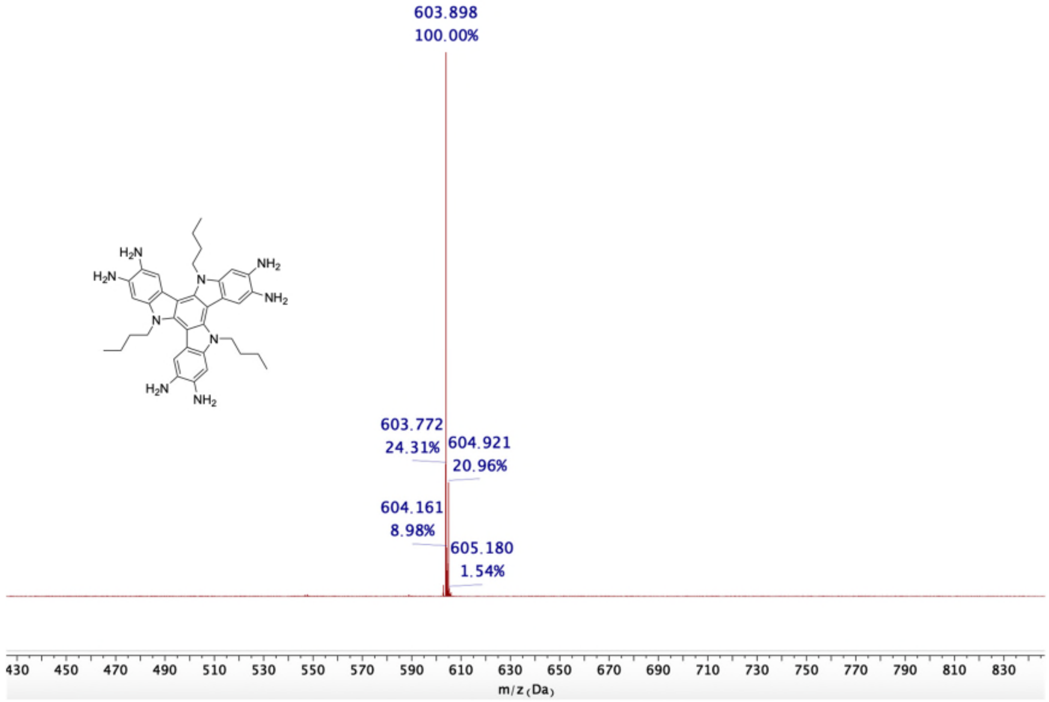
603.898
100.00%
H2N
H2N
NH2
NH2
N
N
N
H2N
NH2
603.772
24.31%
604.921
20.96%
604.161
8.98%
605.180
1.54%
430 450 470 490 510 530 550 570 590 610 630 650 670 690 710 730 750 770 790 810 830
m/z (Da)

Supplementary Figures

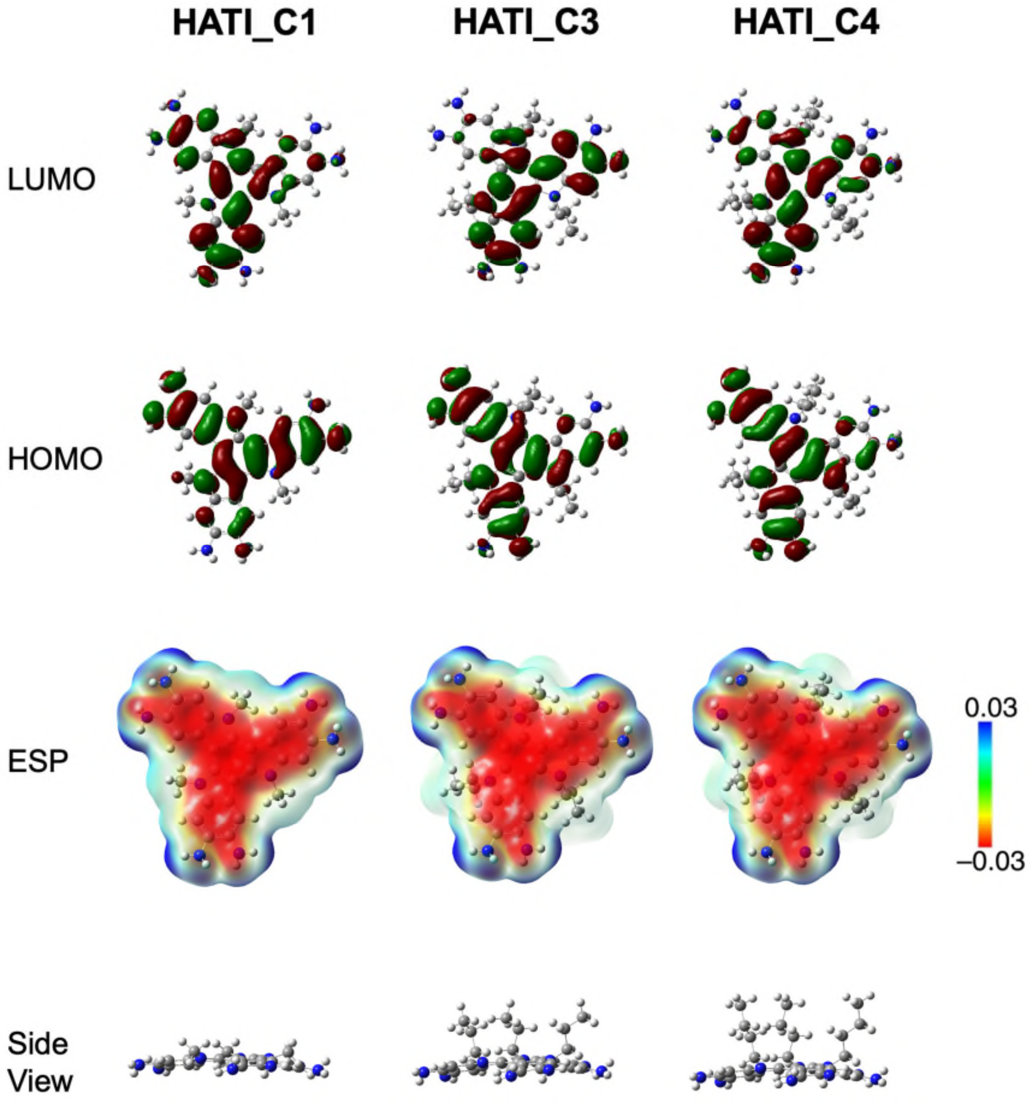

Supplementary Figure 1. The electronic structures (HOMO, LUMO, and electrostatic potential (ESP)) and optimized structures of HATI_CX (X = 1-4) derived from DFT calculations. DFT was performed using Gaussian 09 with the B3LYP functional and 6-311g(d,p) basis set and the Grimme D3 dispersion correction.[16] The alkyl chains with different lengths almost didn't alter the frontier orbital distribution and the charge distribution. But the length of the alkyl chain directly affects its free volume in the relaxed state, so the longer the length (the larger the volume) of the alkyl side chain, the more significant the steric hindrance effect between chains. When the two 2D layers are brought close to each other, the interaction between the alkyl chains will compete with the π-π interaction between the conjugated ligands. Therefore, as the side chain of the ligand gradually grows from methyl to butyl, the interlayer distance gradually increases, which in turn changes the electronic structure and charge transport properties.

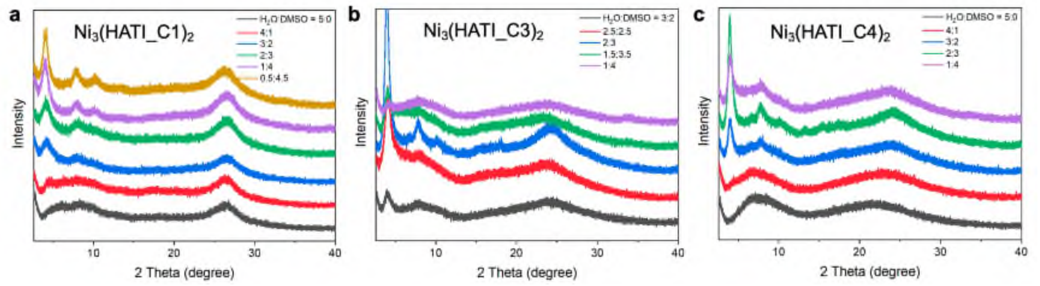

Supplementary Figure 2. Optimization of Ni_3[HATI_CX]$_2$ synthesis. (a) PXRD of Ni_3[HATI_C1]$_2$ synthesized under different H_2O/DMSO ratios. The best condition for Ni_3[HATI_C1]$_2$ is H_2O/DMSO = 0.5:4.5. (b) PXRD of Ni_3[HATI_C3]$_2$ synthesized under different H_2O/DMSO ratios. The best condition for Ni_3[HATI_C3]$_2$ is H_2O/DMSO = 2.0:3.0. (c) PXRD of Ni_3[HATI_C4]$_2$ synthesized under different H_2O/DMSO ratios. The best condition for Ni_3[HATI_C4]$_2$ is H_2O/DMSO = 2.0:3.0. As the side chain increasing, the best results don't need the high ratio good solvent (DMSO). Solubility of ligand plays an important role in the 2D c-MOF synthesis.

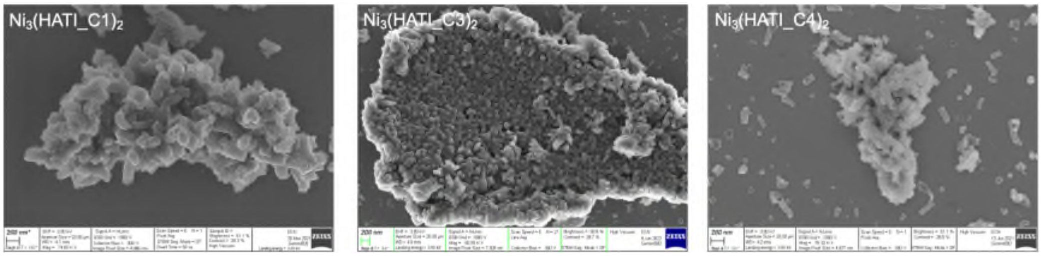

Supplementary Figure 3. SEM images of bulk $Ni_3[HATI\_CX]_2$ MOFs. $Ni_3(HATI\_C1)_2$, $Ni_3(HATI\_C3)_2$, and $Ni_3(HATI\_C4)_2$ powders all show the rod-like crystals

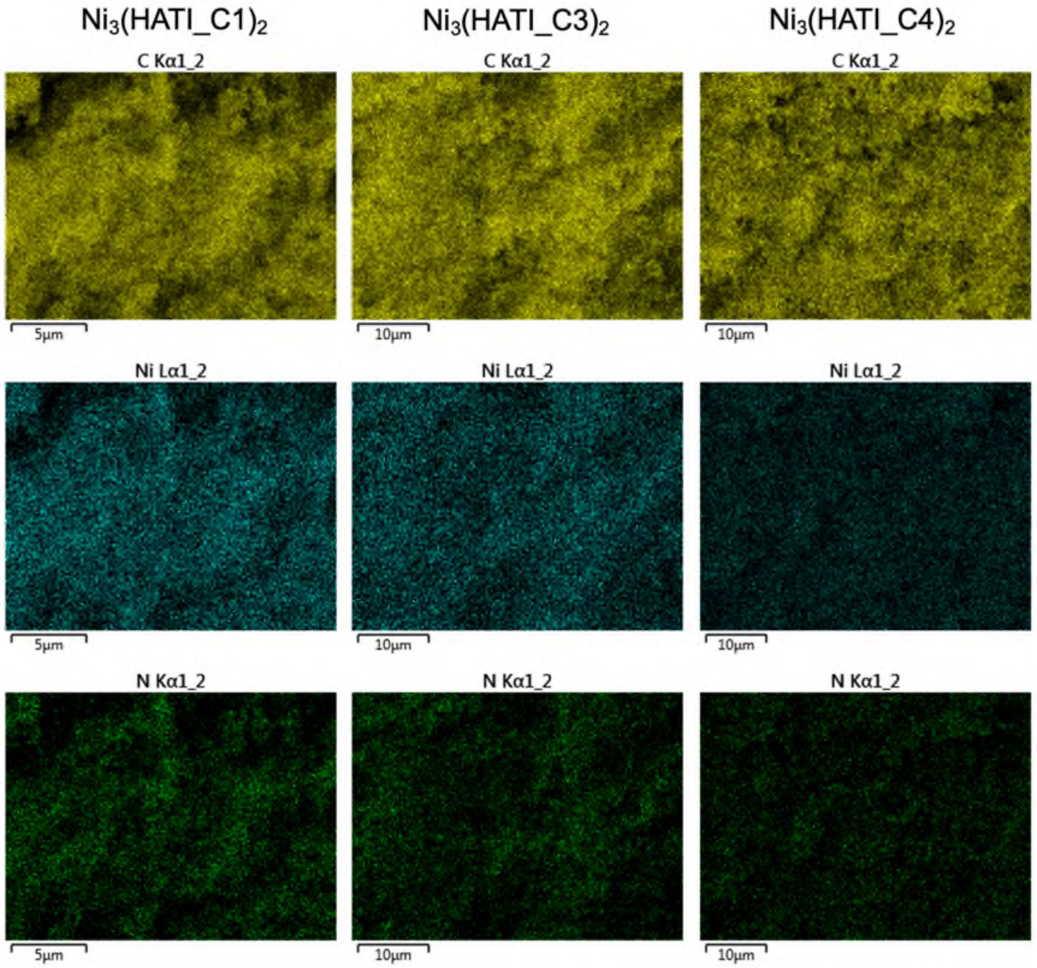

Supplementary Figure 4. Energy dispersive X-ray (EDX) spectroscopy of the elemental mapping images of bulk $Ni_3[HATI\_CX]_2$ MOFs. The uniformly distributed C, Ni, and N elements confirmed the existence of these components.

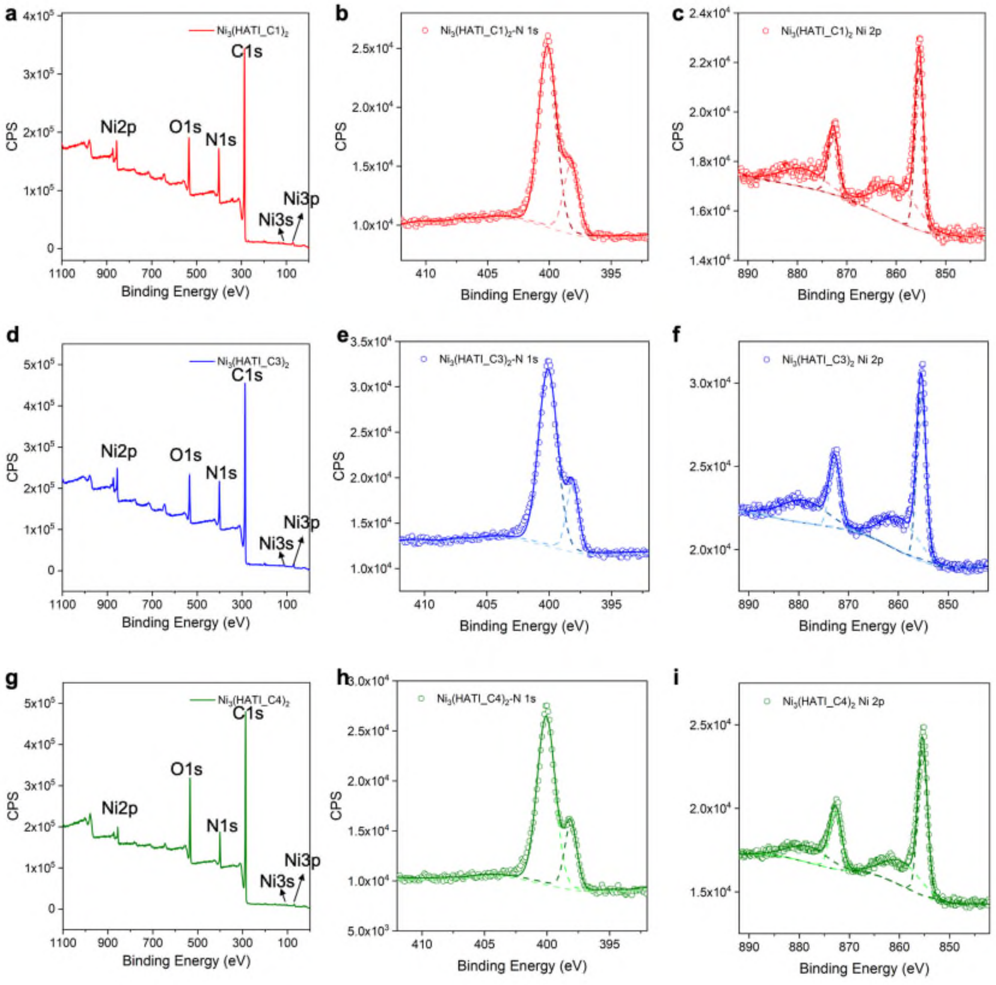

Supplementary Figure 5. XPS measurements of the MOF samples. (a-c) X-ray photoelectron spectroscopy survey spectrum, high-resolution spectrum for N (1s) and Ni (2p) of $Ni_3[HATI\_C1]_2$. (d-f) X-ray photoelectron spectroscopy survey spectrum, high-resolution spectrum for N (1s) and Ni (2p) of $Ni_3[HATI\_C3]_2$. (g-i) X-ray photoelectron spectroscopy survey spectrum, high-resolution spectrum for N (1s) and Ni (2p) of $Ni_3[HATI\_C4]_2$.

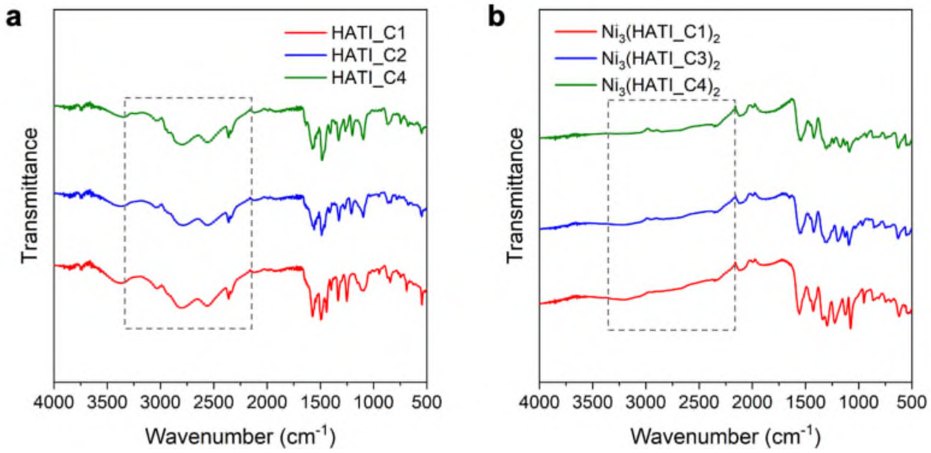

Supplementary Figure 6. FT-IR spectra of ligands and the resultant MOF samples. (a) HATI_CX ligands and (b) $Ni_3[HATI\_CX]_2$ MOFs.

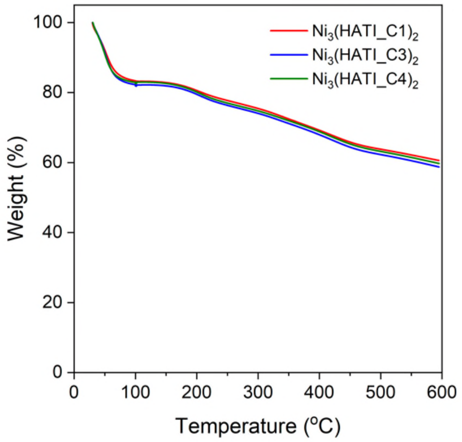

Supplementary Figure 7. Thermo Gravimetric Analysis (TGA) of Ni_3[HATI_CX]$_2$. TGA revealed that all Ni_3[HATI_CX]$_2$ decompose above 200 °C. However, the weight loss before 100 °C of the samples could not be totally excluded. The reason for this phenomenon might be originated from the strong hydrophilicity of the Ni[NH]$_4$ nodes which leads to the rapid water uptake of the samples during the weighing and transfer process. This phenomenon was also found in previous works (*J. Am. Chem. Soc.* **2014**, *136*, 8859; *J. Am. Chem. Soc.* **2017**, *139*, 13608). Therefore, we chose the *in-situ* activation (100 °C for 1 h) during the TGA measurement.

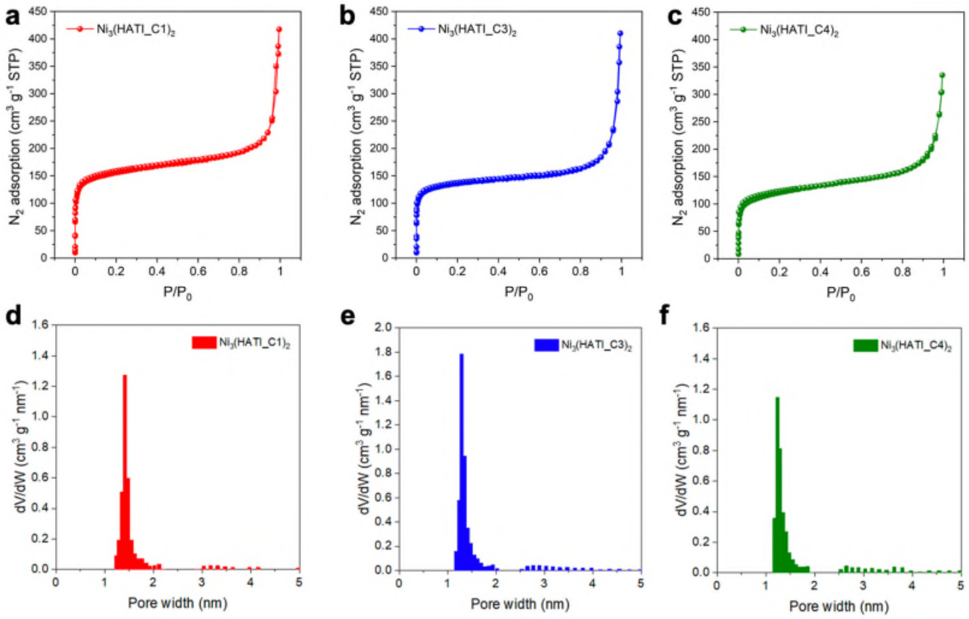

Supplementary Figure 8. Porosity properties of the resultant MOF samples. (a-c) N_2 adsorption/desorption isotherms at 77.3 K of $Ni_3[HATI\_CX]_2$ and (d-f) the corresponding pore size distribution of $Ni_3[HATI\_CX]_2$ (calculated using the QSDFT method). All materials showed the similar pore distribution, suggesting the lengths of alkyl chain had small effects on the pore structures.

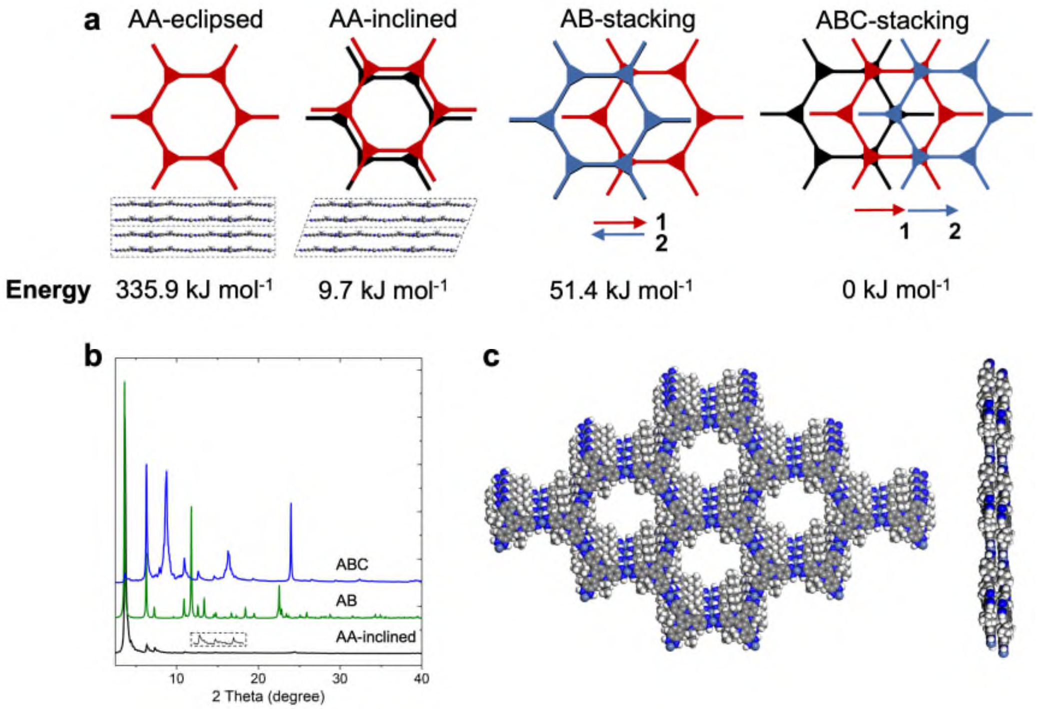

Supplementary Figure 9. DFT calculation of MOF structures. (a) Schematic diagram of four stacking models (AA-eclipsed, AA-inclined, AB, and ABC) of $Ni_3(HATI\_CX)_2$. The dashed line represents the unit cell. (b) Calculated PXRD patterns of $Ni_3(HATI\_CX)_2$ with different stacking modes. (c) The AA-inclined structures of $Ni_3(HATI\_C3)_2$. In order to understand the stacking structures of $Ni_3(HATI\_CX)_2$, we systematically compared the energies of AA-eclipsed, AA-inclined, AB, and ABC stacking. Comparing the relative energies and PXRD patterns, we conclude that $Ni_3(HATI\_CX)_2$ tends to accumulate in the solid state in the AA-inclined stacking mode.

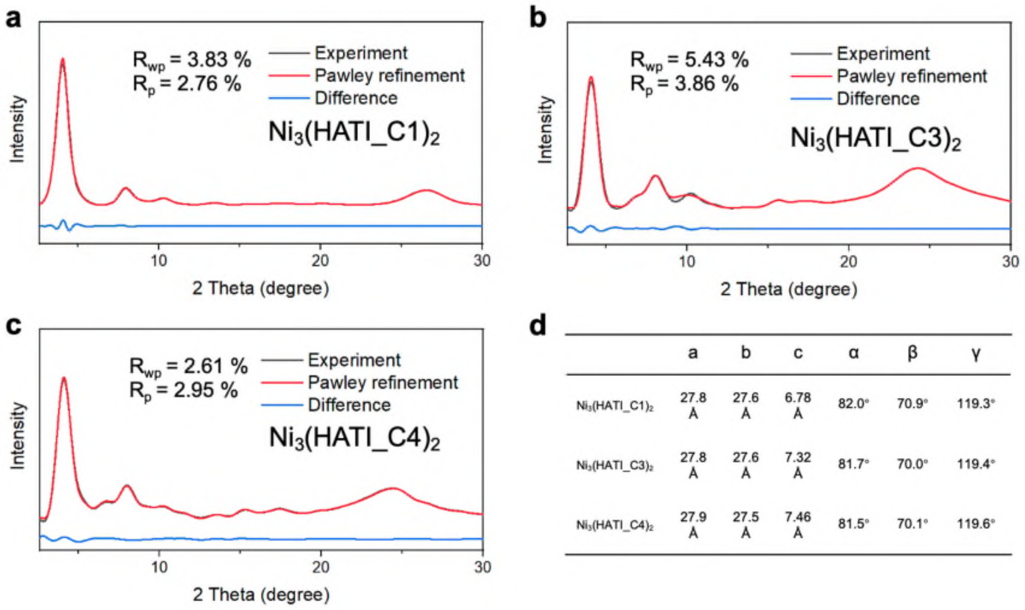

Supplementary Figure 10. Crystalline structure analysis of MOF samples. Pawley refinements for (a) $Ni_3[HATI\_C1]_2$, (b) $Ni_3[HATI\_C3]_2$, and (c) $Ni_3[HATI\_C4]_2$. (d) The detailed results of Pawley refinement for $Ni_3[HATI\_CX]_2$. The final Rwp and Rp values 3.83 % and 2.76 % for $Ni_3(HANT\text{-}C1)_2$, 5.43 % and 3.86 % for $Ni_3(HANT\text{-}C3)_2$, and 2.61 % and 2.95 % for $Ni_3(HANT\text{-}C4)_2$, respectively was obtained after the Pawley refinement.

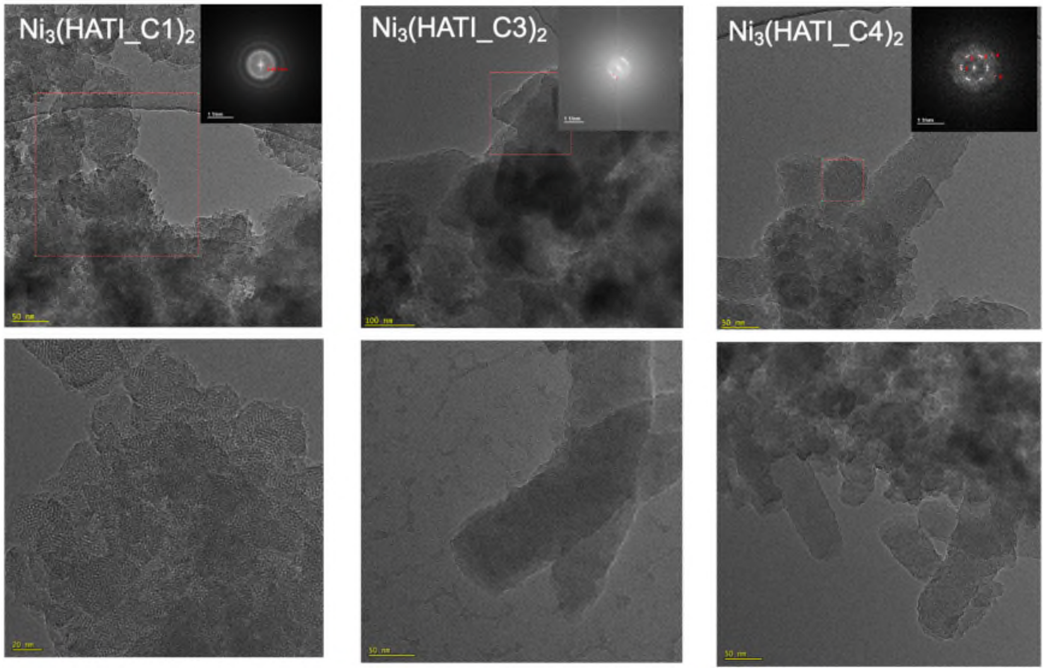

Supplementary Figure 11. HRTEM images of $Ni_3(HATI\_CX)_2$ and the inset shows the FFT analysis of the micrograph. The fast Fourier transform (FFT) analysis of HRTEM images of $Ni_3(HATI\_CX)_2$ presented the honeycomb lattice with the lattice distance of 25.4 Å, which suggested the different length alkyl chain didn’t influence the lattice parameter within 2D plane.

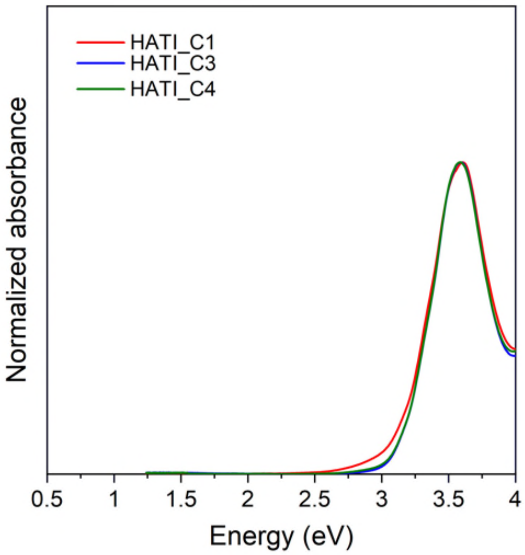

Supplementary Figure 12. Vis−NIR absorption spectroscopy of HATI_CX. All ligands presented an almost identical spectrum with the main absorption peak at 3.6 eV, suggesting that the alkyl chains do not affect the photophysical properties of the aromatic core.

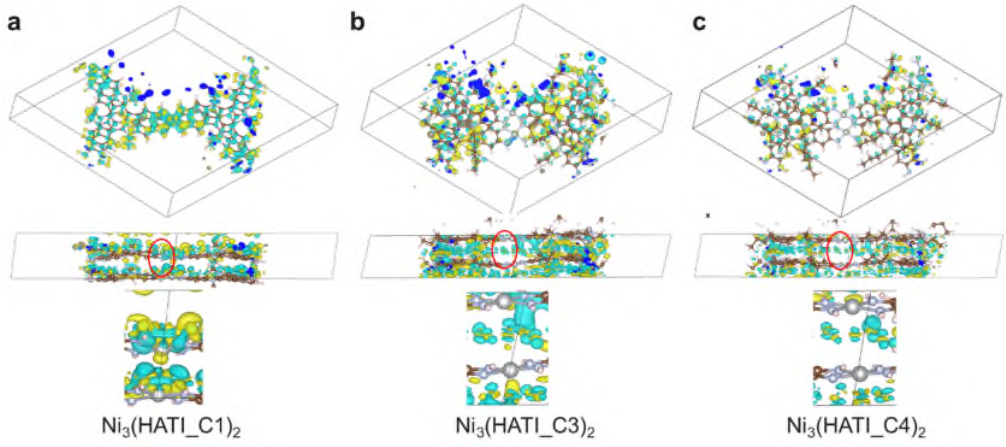

Supplementary Figure 13. Calculation of charge distribution of MOF samples. Top and side view of the charge density difference between layers for a) $Ni_3(HATI\_C1)_2$, b) $Ni_3(HATI\_C3)_2$, and c) $Ni_3(HATI\_C4)_2$. The yellow (cyan) distribution corresponds to charge accumulation (depletion). The isosurface is taken as 5×10^{-3} e/Å^3. The bottom is the enlarged side view of Ni[NH]$_4$ nodes. The electronic coupling of the Ni[NH]$_4$ nodes to Ni[NH]$_4$ nodes gradually decreased as the interlayer distance increasing.

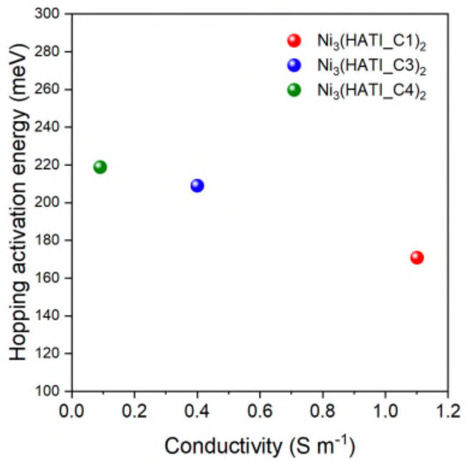

Supplementary Figure 14. The relationship between hopping activation energies and electrical conductivities of $Ni_3(HATI\_CX)_2$. The hopping activation energies of the three materials are positively correlated with the interlayer distance.

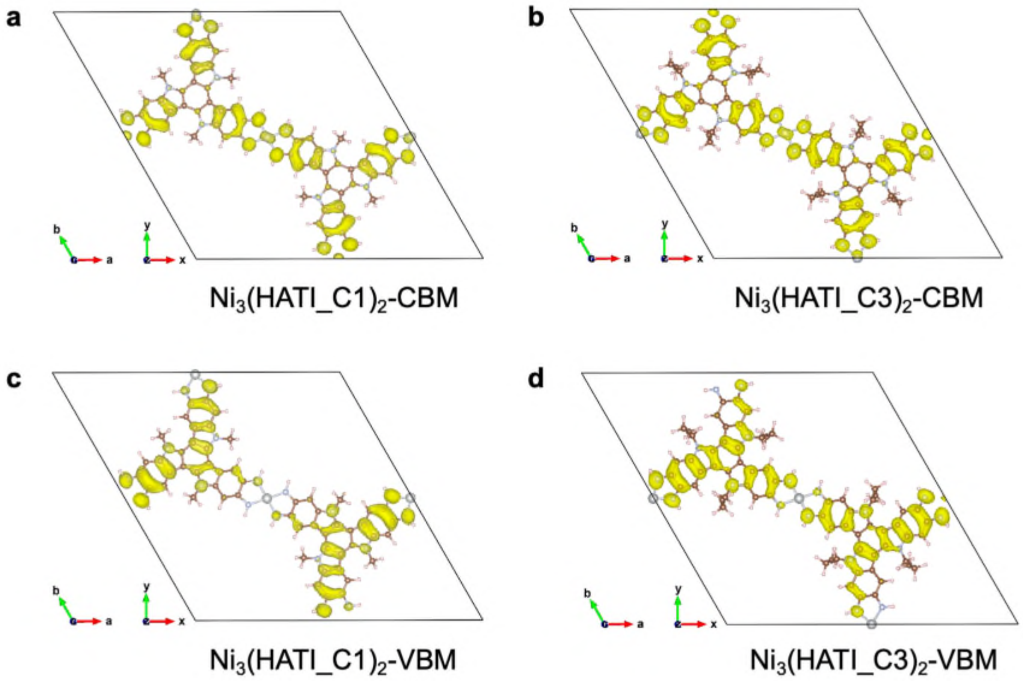

Supplementary Figure 15. The surface charge distribution of Ni_3(HATI_C1)$_2$ and Ni_3(HATI_C3)$_2$. a) the conduction band surface charge distribution and c) the valence band surface charge distribution of Ni_3(HATI_C1)$_2$; b) the conduction band surface charge distribution and d) the valence band surface charge distribution of Ni_3(HATI_C3)$_2$. There is no charge distribution on the alkyl chains, therefore, alkyl chains of different lengths do not significantly alter the charge transport properties within the 2D layer in Ni_3(HATI_CX)$_2$ materials.

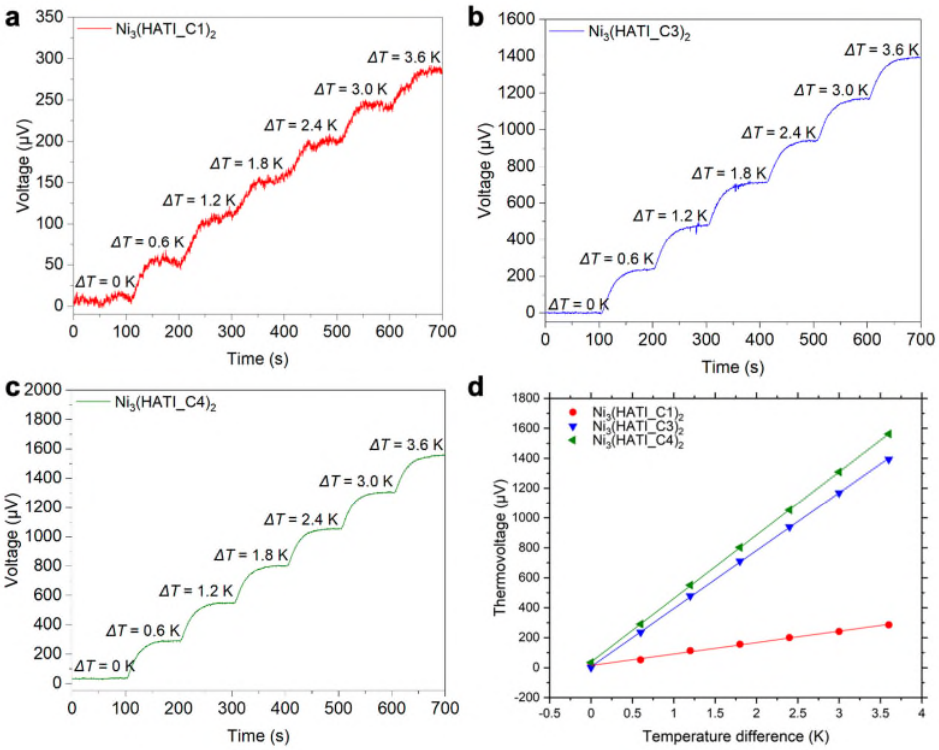

Supplementary Figure 16. Seebeck coefficient measurements. Temperature difference dependent thermal voltage and current in thermal voltage measurement for (a) Ni_3(HATI_C1)$_2$, (b) Ni_3(HATI_C3)$_2$, and (c) Ni_3(HATI_C4)$_2$. (d) The fitting line of temperature difference dependent thermal voltage of Ni_3(HATI_CX)$_2$.

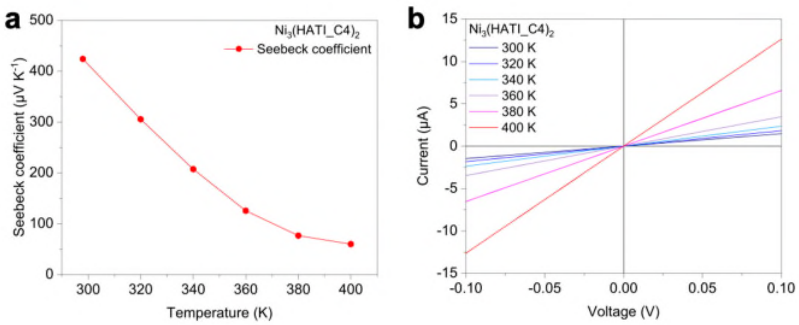

Supplementary Figure 17. Temperature-dependent Seebeck coefficient measurements. (a) Plot of the Seebeck coefficient of $Ni_3(HATI\_C4)_2$ versus temperature. (b) Current-voltage (I-V) curves of $Ni_3(HATI\_C4)_2$ at different temperatures. The Seebeck coefficient $Ni_3(HATI\_C4)_2$ decreases with increasing temperature, and at the same time, the electrical conductivity of the pellet increases significantly. These results are consistent with previous studies and further demonstrate the thermally active charge transport behavior of these semiconductive 2D c-MOFs.

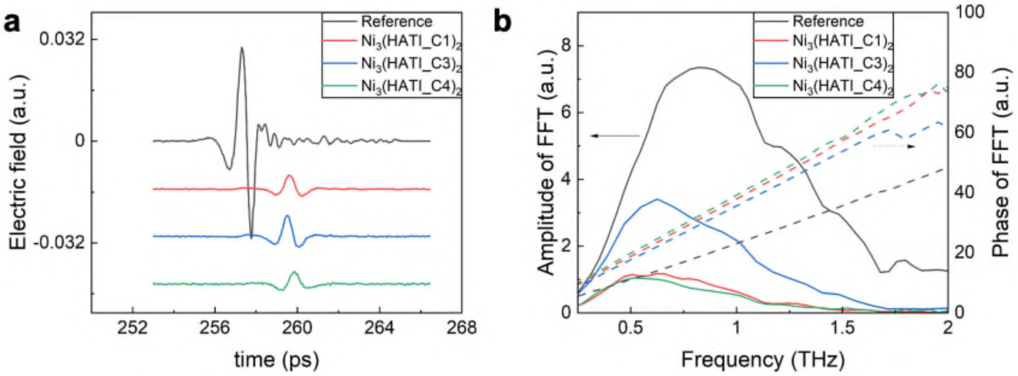

Supplementary Figure 18. THz-TDS measurements. (a) Electric field signals acquired from the reference and from the different samples. (b) Phase information obtained from the FFT of the electric fields.

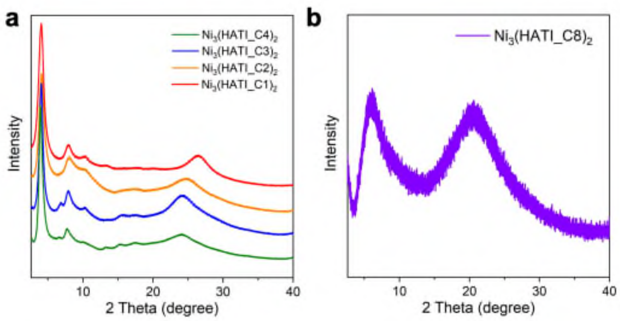

Supplementary Figure 19. Supplementary information on $Ni_3(HATI\_CX)_2$ MOFs substituted with other alkyl side chains. (a) XRD patterns for $Ni_3(HATI\_C1)_2$, $Ni_3(HATI\_C2)_2$, $Ni_3(HATI\_C3)_2$, and $Ni_3(HATI\_C4)_2$. (b) XRD patterns for $Ni_3(HATI\_C8)_2$. The synthesis method of $Ni_3(HATI\_C2)_2$ and $Ni_3(HATI\_C8)_2$ were provided in Section S2. The interlayer distance of $Ni_3(HATI\_C2)_2$ is 3.59 Å, which follows the same trends as other MOFs. However, although we screened many reaction conditions, the crystallinity of $Ni_3(HATI\_C2)_2$ is significantly lower than the other samples, which is revealed by the XRD pattern (Table S4). As is known, the electrical properties of conductive MOFs largely depend on crystallinity. Therefore, to avoid confusion, we didn't involve $Ni_3(HATI\_C2)_2$ in the main text. When we synthesized $Ni_3(HATI\_CX)_2$ MOFs with ligands substituted with longer alkyl chains (e.g., -octyl, X = 8), we also observed a further increase in interlayer spacing (4.37Å). However, the crystallinity and stacking mode of $Ni_3(HATI\_C8)_2$ have changed significantly (the (100) peak shifted), which may be caused by the overcrowding of the long alkyl chains in the pores. As is known, both the crystallinity and the stacking mode of MOFs have a direct influence on the electrical properties. Therefore, to avoid confusion, we didn't involve $Ni_3(HATI\_C8)_2$ in the main text.

Supplementary Tables

Supplementary Table 1. Summary of DFT optimized structure data of $Ni_3(HATI\_CX)_2$ MOFs.

	a	b	c	α	β	γ
**$Ni_3(HATI\_C1)_2$**	28.0 Å	27.9 Å	6.82 Å	81.5°	70.5°	119.2°
**$Ni_3(HATI\_C3)_2$**	28.0 Å	27.9 Å	7.35 Å	81.2°	69.4°	119.6°
**$Ni_3(HATI\_C4)_2$**	28.0 Å	27.9 Å	7.44 Å	81.0°	69.1°	119.7°

Supplementary Table 2. Summary of the room temperature thermoelectric properties of reported MOFs.

MOF Materials	σ (S/m)	S (μV/K)	*PF* (nW m^{-1} K^{-2})	P or N Type	Reference
$Ni_3(HATI\_C3)_2$	0.45	389	68	P	This work
$Ni_3(HATI\_C4)_2$	0.09	424	16.2	P	This work
$Ni_3(HATI\_C1)_2$	1.1	76.3	6.4	P	This work
a-Pt-HITP	32.7	37.7	46	P	*ACS Appl. Mater. Interfaces* **2021**, *13*, 52055.
Zn-HAB	0.086	200	3.4	P	*J. Am. Chem. Soc.* **2020**, *142*, 20531.
Cu_3BTC_2@TCNQ	0.41	375	57.6	P	*Adv. Mater.* **2015**, *27*, 3453.
$Cu_3(HHTP)_2$	0.23	-7.24	3.15	N	*J. Mater. Chem. A* **2020**, *8*, 13197.
$Ni_3(HITP)_2$	5880	-11.9	833	N	*Joule* **2017**, *1*, 168.

Supplementary Table 3. The comparison of electrical conductivities of $Ni_3(HATI\_CX)_2$ at different activation treatment.

	Without activation	Activation at 100 °C overnight	Activation at 150 °C, 2h
**$Ni_3(HATI\_C1)_2$**	1.39 S m^{-1}	1.12 S m^{-1}	1.12 S m^{-1}
**$Ni_3(HATI\_C3)_2$**	0.57 S m^{-1}	0.43 S m^{-1}	0.42 S m^{-1}
**$Ni_3(HATI\_C4)_2$**	0.11 S m^{-1}	0.085 S m^{-1}	0.086 S m^{-1}

The electrical conductivities of $Ni_3(HATI\_CX)_2$ show the same variation trends: the electrical conductivities gradually decrease as the alkyl chain length increases. After the activation treatment, the electrical conductivities of samples are slightly lower than the samples without activation. The different activation temperature (100 °C *vs.* 150 °C) has almost no influence on the electrical behaviors of the $Ni_3(HATI\_CX)_2$ system.

Supplementary Table 4. The comparison of crystallinity of $Ni_3(HATI\_CX)_2$

	Half width of (100) peak	Average size of nanocrystals
**$Ni_3(HATI\_C1)_2$**	0.559 °	14.1 nm
**$Ni_3(HATI\_C2)_2$**	0.819 °	9.6 nm
**$Ni_3(HATI\_C3)_2$**	0.541 °	14.5 nm
**$Ni_3(HATI\_C4)_2$**	0.534 °	14.7 nm

Supplementary References:

1. Cocker, T. L. et al. Microscopic origin of the Drude-Smith model. *Phys. Rev. B* **96**, 205439 (2017).
2. Smith, N. V. Classical generalization of the Drude formula for the optical conductivity. *Phys. Rev. B* **64**, 155106 (2001).
3. Yang, C. Y. et al. A high-conductivity n-type polymeric ink for printed electronics. *Nat. Commun.* **12**, 2354 (2021).
4. Sun, L. et al. A Microporous and Naturally Nanostructured Thermoelectric Metal-Organic Framework with Ultralow Thermal Conductivity. *Joule* **1**, 168-177 (2017).
5. Porezag, D., Frauenheim, T., Köhler, T., Seifert, G. & Kaschner, R. Construction of tight-binding-like potentials on the basis of density-functional theory: Application to carbon. *Phys. Rev. B* **51**, 12947-12957 (1995).
6. te Velde, G. et al. Chemistry with ADF. *J. Comput. Chem.* **22**, 931-967 (2001).
7. Hermoso-Diaz, I. A. et al. Electrochemical and quantum chemical assessment of linoleic acid as a corrosion inhibitor for carbon steel in sulfuric acid solution. *J. Mol. Struct.* **1197**, 535-546 (2019).
8. Rappe, A. K., Casewit, C. J., Colwell, K. S., Goddard, W. A. & Skiff, W. M. UFF, a full periodic table force field for molecular mechanics and molecular dynamics simulations. *J. Am. Chem. Soc.* **114**, 10024-10035 (1992).
9. Blum, V. et al. Ab initio molecular simulations with numeric atom-centered orbitals. *Comput. Phys. Commun.* **180**, 2175-2196 (2009).
10. Heyd, J., Scuseria, G. E. & Ernzerhof, M. Hybrid functionals based on a screened Coulomb potential. *J. Chem. Phys.* **118**, 8207-8215 (2003).
11. Tkatchenko, A., DiStasio, R. A., Car, R. & Scheffler, M. Accurate and Efficient Method for Many-Body van der Waals Interactions. *Phys. Rev. Lett.* **108**, 236402 (2012).
12. Kresse, G.; Furthmüller, J. Efficient iterative schemes for ab initio total-energy calculations using a plane-wave basis set. *Phys. Rev. B* **54**, 11169-11186 (1996).
13. Kresse, G.; Furthmuller, J. Efficiency of ab-initio total energy calculations for metals and semiconductors using a plane-wave basis set. *Comput. Mater. Sci.* **6**, 15-50 (1996).
14. Perdew, J. P.; Burke, K.; Ernzerhof, M. Generalized gradient approximation made simple. *Phys. Rev. Lett.* **77**, 3865-3868 (1996).
15. Grimme, Stefan, Stephan Ehrlich, and Lars Goerigk. Effect of the damping function in dispersion corrected density functional theory. *J. Comput. Chem.* **32**, 1456-1465 (2011).
16. Gaussian 09, Revision C.01, M. J. Frisch, G. W. Trucks, H. B. Schlegel, G. E. Scuseria, M. A. Robb, J. R. Cheeseman, G. Scalmani, V. Barone, B. Mennucci, G. A. Petersson, H. Nakatsuji, M. Caricato, X. Li, H. P. Hratchian, A. F. Izmaylov, J. Bloino, G. Zheng, J. L. Sonnenberg, M. Hada, M. Ehara, K. Toyota, R. Fukuda, J. Hasegawa, M. Ishida, T. Nakajima, Y. Honda, O. Kitao, H. Nakai, T. Vreven, J. A. Montgomery Jr., J. E. Peralta, F. Ogliaro, M. Bearpark, J. J. Heyd, E. Brothers, K. N. Kudin, V. N. Staroverov, T. Keith, R. Kobayashi, J. Normand, K. Raghavachari, A. Rendell, J. C. Burant, S. S. Iyengar, J. Tomasi, M. Cossi, N. Rega, J. M. Millam, M. Klene, J. E. Knox, J. B. Cross, V. Bakken, C. Adamo, J. Jaramillo, R. Gomperts, R. E. Stratmann, O. Yazyev, A. J. Austin, R. Cammi, C. Pomelli, J. W. Ochterski, R. L. Martin, K. Morokuma, V. G. Zakrzewski, G. A. Voth, P. Salvador, J. J. Dannenberg, S. Dapprich, A. D. Daniels, O. Farkas, J. B. Foresman, J. V. Ortiz, J. Cioslowski, D. J. Fox, Gaussian, Inc., Wallingford CT, 2010.
